# Designing logical codon reassignment – Expanding the chemistry in biology†
†Electronic supplementary information (ESI) available: A comprehensive table of the UAAs incorporated to date (also summarized in Table 1), their reported/potential uses, and the required mutations in the aaRS to allow their uses. See DOI: 10.1039/c4sc01534g


**DOI:** 10.1039/c4sc01534g

**Published:** 2014-07-14

**Authors:** Anaëlle Dumas, Lukas Lercher, Christopher D. Spicer, Benjamin G. Davis

**Affiliations:** a Chemistry Research Laboratory , Department of Chemistry , University of Oxford , Mansfield Road , Oxford , OX1 3TA , UK . Email: Ben.Davis@chem.ox.ac.uk

## Abstract

Over the last decade, the ability to genetically encode unnatural amino acids (UAAs) has evolved rapidly. The programmed incorporation of UAAs into recombinant proteins relies on the reassignment or suppression of canonical codons with an amino-acyl tRNA synthetase/tRNA (aaRS/tRNA) pair, selective for the UAA of choice. In order to achieve selective incorporation, the aaRS should be selective for the designed tRNA and UAA over the endogenous amino acids and tRNAs. Enhanced selectivity has been achieved by transferring an aaRS/tRNA pair from another kingdom to the organism of interest, and subsequent aaRS evolution to acquire enhanced selectivity for the desired UAA. Today, over 150 non-canonical amino acids have been incorporated using such methods. This enables the introduction of a large variety of structures into proteins, in organisms ranging from prokaryote, yeast and mammalian cells lines to whole animals, enabling the study of protein function at a level that could not previously be achieved. While most research to date has focused on the suppression of ‘non-sense’ codons, recent developments are beginning to open up the possibility of quadruplet codon decoding and the more selective reassignment of sense codons, offering a potentially powerful tool for incorporating multiple amino acids. Here, we aim to provide a focused review of methods for UAA incorporation with an emphasis in particular on the different tRNA synthetase/tRNA pairs exploited or developed, focusing upon the different UAA structures that have been incorporated and the logic behind the design and future creation of such systems. Our hope is that this will help rationalize the design of systems for incorporation of unexplored unnatural amino acids, as well as novel applications for those already known.

## Abstract 2

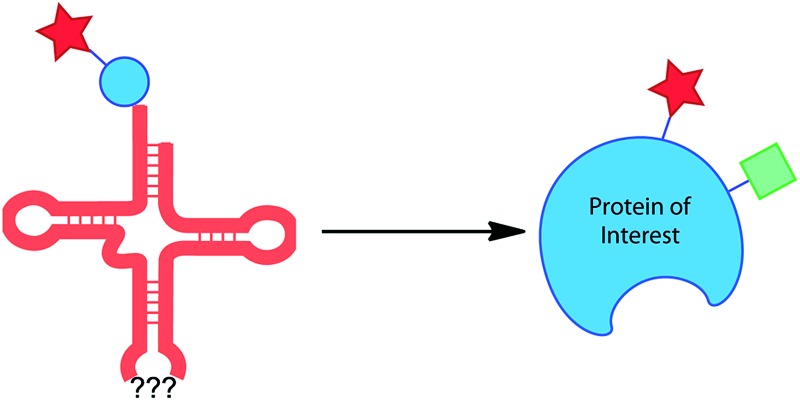
This review rationalizes the varied designs of systems for incorporation of UAAs into proteins *via* canonical codons.

## Introduction

**Fig. 1:**
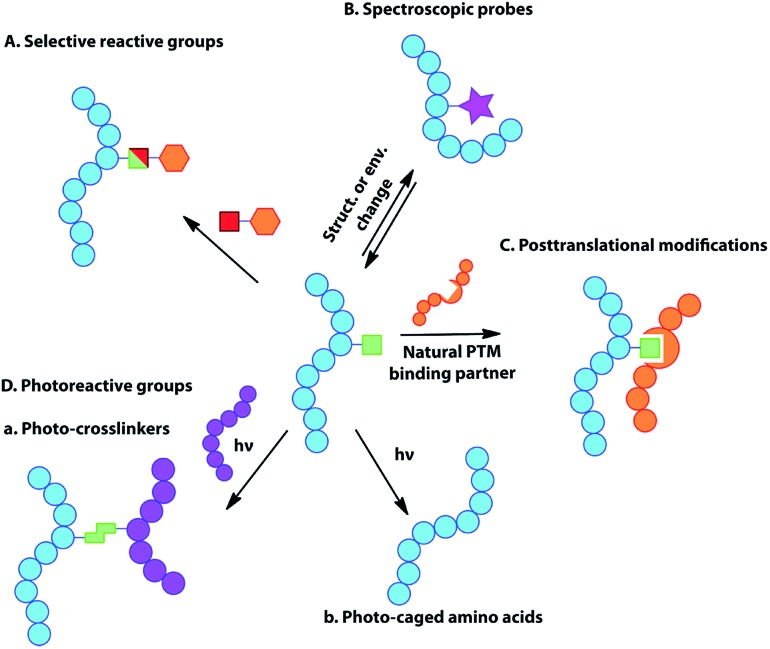
Schematic representation of the major uses of genetically encoded UAA side chains, including selectively reactive groups, spectroscopic probes, natural post-translational modifications or mimics and photoreactive-groups (photo-crosslinkers and photo-caged amino acids).

While DNA and RNA make up the essential genetic information needed to create a living cell,^1^ it is the proteins that they code for that are the ‘workhorses’ of the cell, playing a key role in virtually every biological process and structure.^2^ The vast diversity of proteins is even more striking, given that they are typically made of just 20 natural amino acid building blocks, with an even more limited set of chemical functionalities. While our understanding of cellular biology has undoubtedly expanded vastly over the past century, more questions that cannot be answered *via* the techniques currently available to researchers emerge with each new discovery. New tools are required to investigate and manipulate proteins in both an intra and extra-cellular context.^3^ The ability to incorporate unnatural amino acids (UAAs) into proteins offers the potential to study, alter, or even improve upon protein activity and function through molecular ingenuity, both *in vitro* and *in vivo*.^4^ Such UAAs may modulate enzyme activity, offer unique reactive handles for further modification, act as spectral or imaging probes, or simply offer a novel functional structure or natural post-translational modification (PTM) or mimic (Fig. 1).^5^

Many techniques have emerged for incorporating UAAs into proteins in a variety of organisms over the past few decades.^6^ These include expansion of the genetic code by codon reassignment and suppression, of both sense and non-sense codons^7^ (see Table 1). Codon reassignment can allow the site-specific installation of an UAA into a protein of choice. In general, this technique relies on the exploitation or development of a selective amino-acyl tRNA synthetase/tRNA (aaRS/tRNA) pair, specific for the UAA of choice and the codon to be reassigned. Greater flexibility comes from an aaRS/tRNA pair that does not cross-react with the existing host cell’s pool of synthetases, tRNAs and translational machinery, but that is recognised by the host ribosome; this mutually compatible (or ‘orthogonal’) pair results in incorporation of the UAA. The most commonly used systems have relied upon the reassignment of ‘non-sense’ codons, as codons that are not used for amino acids by the endogenous systems, particularly (but not exclusively) the amber-stop codon, TAG. This has resulted in the incorporation of a diverse range of UAAs not only in prokaryote^7a,8^ and eukaryote^7b,9^ cell culture, but more recently in multicellular organisms and even animals.^10^ Other systems based on the reassignment of the ochre and opal stop codons,^11^ in addition to the use of quadruplet codons have also been reported (see later section).^12^ It is now also possible to consider their combined use for the incorporation of multiple distinct UAAs into a single protein of choice.^11^ Of course, competition with the host cell termination machinery at stop codons offers a significant drawback to ‘non-sense’ suppression, commonly resulting in lower yields and truncated protein products. The use of ‘sense’ codon reassignment offers a potential solution to this problem since it is a system with inherent translational efficiency. While such technical hurdles still exist, the field of UAA incorporation is still only in its infancy and the potential power of diverse, near unlimited chemical functionality in biology offers a truly exciting prospect (see ‘Outlook’ section).

**Table 1:** Unnatural amino acids that have been genetically encoded in proteins to date. The square's colours represent the organism(s) in which these UAAs have been encoded, the circle's colours the aaRS/tRNA pair(s) used for incorporation and the triangle's colours the application(s) of the corresponding UAA in proteins

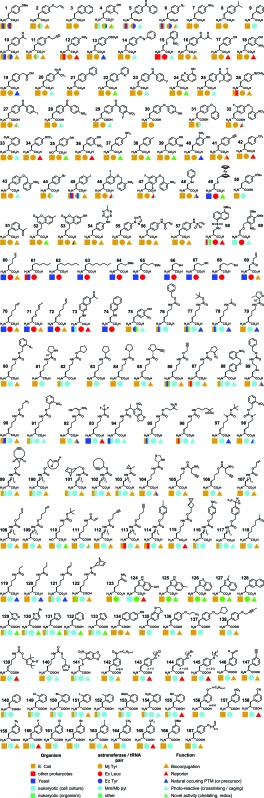

In this review, we will focus on the site-specific incorporation of unnatural amino acids *via* codon reassignment. We aim not to describe the theory and techniques behind this technology, as this has been excellently reviewed elsewhere. Though a brief historical perspective will be given, the reader is directed to reviews by Liu,^5^ Young^4^ and Davis^13^ for a more detailed description. Rather, we hope to provide an overview of the structures and functionalities that have been incorporated, based on the aaRS/tRNA system that has been utilised (and hence the codons that have been reassigned), to provide a reference for those who wish to incorporate novel UAAs or use those that have already been described. We also provide a comprehensive table of all the UAAs incorporated to date along with details of their applications to date (see Table 1), as well as details of the mutations required to generate the requisite aaRS (see the ESI†). In particular, we wish to highlight the unique *chemistries* of the available UAAs, to aid researchers in developing novel applications for a field that has the potential to revolutionize the biological sciences in a way that site-directed mutagenesis did before it,^14^ perhaps more so.

## Applications

The genetic encoding of UAAs *via* codon reassignment offers the advantage of site-specificity, to yield homogenously modified proteins substituted with a wide variety of otherwise hard to introduce functional groups. Since incorporation uses the host cell's own translational machinery, the modified proteins offer the advantage of being applicable to the study of intracellular biological processes in the very cellular system that produced them. To date, over 150 different UAAs have been incorporated into proteins. These have been used as tools for a number of applications including the study of protein–protein interactions, protein localization, enzymatic activity and cellular signalling, while also providing handles for protein imaging and spectroscopy. The applications of genetically-encoded UAAs have been extensively reviewed elsewhere,^13,15^ but a summary of the major uses is briefly given here.

### Selectively reactive groups (Fig. 1A)

The introduction of uniquely reactive, non-natural chemical groups provides a powerful tool for site-selective labelling and alteration of proteins, through the use of complementarily selective reactions. To date, a large number of biocompatible and selective (so-called ‘bioorthogonal’) reactions have been developed, including Cu(i)-catalyzed or “copper-free” alkyne–azide triazole-forming reactions, the Staudinger ligation, inverse-electron-demand Diels–Alder (IEDDA) reactions between tetrazines and strained alkenes, “photo-click” chemistry between tetrazoles and alkenes, or metal-mediated processes such as olefin metathesis and Suzuki–Miyaura or Sonogashira cross-couplings (for recent reviews, see ref. 16). The genetic encoding of most of the corresponding reaction partners into proteins provides chemical ‘tags’ that may be addressed selectively for site-specific labelling.^17^ Such reactive handles are particularly useful tools for the preparation of protein conjugates with unprecedented control over structure and homogeneity.

In addition to advantageous applications in the development of protein therapeutics, site-specific protein labelling has also played a significant role in investigating a number of biological pathways (for reviews see ref. 15*b*, 16*a*, 16*c* and 18). Such chemoselective reactions are particularly important in enabling “tag-and-modify” approaches for the selective conjugation of proteins with entities that cannot otherwise be directly encoded, such as large posttranslational modifications (or their mimics), spectroscopic probes or other biomolecular moieties.^17*a*^

### Spectroscopic probes (Fig. 1B)

The introduction of spectroscopic probes into proteins can provide useful information regarding protein structure, conformation, localization and intermolecular interactions, allowing the dissection of many complex biological processes. These spectroscopic probes, inserted directly into the protein backbone, may act as reporters of change in chemical environment occurring at the amino acid residue level. Examples of such spectroscopically-active amino acid side chains include fluorophores for fluorescent spectroscopy, spin labels for NMR and EPR, heavy atoms for X-ray crystallography or amino acids displaying unique infrared absorption properties (for reviews see ref. 19). Unfortunately, the genetic encoding of many spectroscopic probes is often limited by their complex structure and large size and it may therefore be preferable to introduce them *via* chemo-selective modification at a genetically-encoded chemical handle (‘tag’) (see previous section).

### Post-translational modifications or mimics (Fig. 1C)

During their life-time, most proteins *in vivo* undergo some form of chemical or enzymatic modification after translation, such as phosphorylation, sulfation, nitration, glycosylation, methylation, acetylation, ubiquitination or lipidation of the amino acid side chains. Such modifications are important in modulating protein function, interactions with binding partners, signal transduction and as a trigger for a number of cellular events. However, the elucidation of their exact roles and functions remains challenging, partly due to the difficulty in preparing homogenously-modified proteins, especially if the modified amino acid is present in multiple copies in the protein of interest. The genetic encoding of post-translational modifications or their structural mimics enables the selective and site-specific introduction of the modified residue in the protein. This provides homogenously-modified products that are highly valuable in elucidating the biological role of the respective modification. For example, lysine residues are known to undergo various modifications responsible for the regulation of a number of biological processes, notably in the context of histones.^20^ The development of selective PylRS/tRNA pairs has readily enabled the genetic encoding of lysine residues bearing natural modifications (due in part to the substructure resemblance of Pyl to Lys), providing valuable tools for the investigation of the ‘histone code’. Other areas such as the study of protein sulfation and phosphorylation, through the incorporation of sulfotyrosine^21^ and phosphoserine^22^ respectively, and oxidative damage^23^ have also been aided by codon reassignment.

### Photoreactive groups (Fig. 1D)

A number of photoreactive amino acids have been encoded in proteins. These can be broadly classified into two groups:

### Photo-crosslinkers

Photo-crosslinkers are able to rapidly form covalent bonds to organic molecules in their close proximity at the time of irradiation. Photo-crosslinking amino acids are therefore powerful tools for mapping biomolecular interactions *in vivo* and have been used extensively to probe protein–protein or nucleic acid–protein interactions and ligand binding. One major advantage offered by genetically-encoded photo-crosslinkers is their ability to provide positional and therefore structural information on protein complexes in their natural environment. The formation of a covalent bond between the two interacting partners allows the study of weak, transient or pH-dependent interactions that may be lost in non-covalent methods.

### Photo-caged amino acids

When site-specifically incorporated into a protein, the protection of a particular amino acid with a photo-labile protecting group (photo-cage) can temporarily block a specific protein function. The interactions of this amino acid with its environment and therefore protein function can then be restored by light irradiation. This enables rapid and specific protein activation inside living cells in order to facilitate the study of the biological consequences.

### Miscellaneous

The incorporation of UAAs can be exploited to generate novel protein properties. A few examples involve the introduction of redox-active, metal-chelating or stabilizing/destabilizing cores based on the introduction of large hydrophobic or aromatic scaffolds. As a result of such incorporations, stimulation of potent immune responses has been reported but one can envisage diverse applications including, *e.g.*, the creation of *de novo* metalloenzymes.

## Non-sense suppression with orthogonal aaRS/tRNA pairs for genetic encoding of unnatural amino acids

Of the 64 naturally-occurring triplet codons, there are 3 which lack a corresponding tRNA capable of adding an amino acid to a growing peptide chain.§
§A brief note on nomenclature: Throughout this review, we will often refer to different amino acyl synthetase–tRNA pairs in shorthand. This will be comprised of the abbreviated name of the organism from which the pair is derived (*e.g. M. jann*), the 3 letter code for the natural amino acid substrate from which it is derived (*e.g.* Tyr), which will proceed the synthetase (RS), followed by the tRNA with its anticodon given in subscript (*e.g.* CUA). So for example, the tyrosyl synthetase–tRNA pair for amber suppression from the archaebacteria *Methanococcus jannaschii* would become *M. jann* TyrRS/tRNA_CUA_. Colloquially, this may also be referred to as *Mj-Tyr*. These ‘stop’- or ‘non-sense’-codons, TAG (amber), TAA (ochre) and TGA (opal) instead result in termination of the translation process *via* the recruitment of release factors.^24^ As such, they are ideal candidates for re-assignment *via* the design of a suitable suppressor-tRNA, particularly the amber codon TAG, the rarest of the 3 codons in *E. coli*.^25^ Re-assignment effectively leads to an incorporation system that is in direct competition with translational termination by the host machinery. This was first achieved through the use of a chemically acylated TAG-specific tRNA by Noren *et al.* in a cell free system and subsequently in *Xenopus* oocytes by Nowak *et al.* ^26^ Whilst allowing the site-specific incorporation of a large number of UAAs, the need to microinject the chemically-acylated tRNA limits the applicability to large cells such as *Xenopus* oocytes and leads to very small quantities of protein.^27^ In order to achieve general applicability in cells, it is necessary to identify an orthogonal aaRS/tRNA pair that can be uniquely acylated in the organism of choice by an engineered aaRS.^28^ Liu *et al.* reported the first use of an encoded suppressor-tRNA for incorporation of a natural glutamine amino acid *in vivo*.^29^ However, it was not until the discovery of a yeast aaRS/tRNA pair from *S. cerevisiae* that was completely orthogonal to the translational machinery of *E. coli*, that the prospect of incorporating an unnatural amino acid by stop-codon suppression *in vivo* became achievable.^30^

The idea of transferring an aaRS/tRNA pair from another kingdom into the organism of interest would provide the basis for many of the subsequent discoveries in the field, since recognition sequences are often species specific. Indeed, the first incorporation of a UAA *in vivo via* codon reassignment was achieved by Wang *et al.* in *E. coli*, with an orthogonal TyrRS/tRNA_CUA_ pair from the archaebacteria *Methanococcus jannaschii*.^7*a*^ *p*-Methoxyphenylalanine (1) was shown to be incorporated site-specifically into a model protein, with excellent selectivity and fidelity in response to the amber stop codon. This ‘*Mj-Tyr*’ aaRS/tRNA pair has since gone on to be widely used for the incorporation of a wide-range of UAAs, with the development of efficient +ve/–ve selection protocols allowing the rapid screening of UAA specificities from large libraries of mutants (usually 10^7^–10^8^).^31^

The first example of UAA codon reassignment in eukaryotic cells was demonstrated by Sakamoto *et al.* utilising an *E. coli* TyrRS/*B. stearothermophilus* tRNA_CUA_ pair to incorporate 3-iodotyrosine (**4**), albeit with low fidelity.^9*a*^ Chin *et al.* subsequently demonstrated the use of an *E. coli* TyrRS/tRNA_CUA_ pair for efficient-suppression with a number of UAAs in *S. cerevisiae*. Again, an efficient selection system allowed selectivity screening, but efficiency was limited by the slow growth rate of yeast compared to bacteria.^7*b*^ In this system, the rational design of eukaryotic promoter sequences was essential for effective incorporation.

More recently, the pyrrolysine (Pyl) pairs of *Methanosarcina barkeri* and *Methanosarcina mazei* have emerged as leading candidates in the quest to find a broader aaRS/tRNA pair.^32^ Unlike other pairs which have engineered codon-specificities, pyrrolysine aaRS/tRNA_CUA_ pairs occur naturally in some methanogenic archaea as amber-suppressors. These pairs have been shown to be selective in both prokaryotic and eukaryotic systems, with both wild-type (wt) and mutant synthetases being tolerant of a wide range of UAA structures, allowing their use in a diverse range of labelling and functional studies. Indeed, more recently the group of Chin have demonstrated that an ‘*Mb-Pyl*’ pair can be used for amber suppression in multicellular animals in an impressive demonstration of the power of stop-codon reassignment.^10^

While suppression of the amber-codon has been most widely investigated, the development of pairs for the suppression of both ochre and opal-codons have also been reported. While more prevalent in the genome, potentially leading to increased toxicity with suppression, their use may allow the incorporation of multiple distinct UAAs into the same protein, as achieved by the groups of Liu and Schultz.^11,33^ A further development has seen the decoding of quadruplet codons and orthogonal ribosomes, theoretically offering a large number of new unassigned codons and the possibility to develop a fully orthogonal genetic code in the cell (see ‘Quadruplet codon suppression’ section).^12^ However, to date this approach is still limited by availability of mutually orthogonal aaRS/tRNA_NNNN_ pairs.

**Fig. 2:**
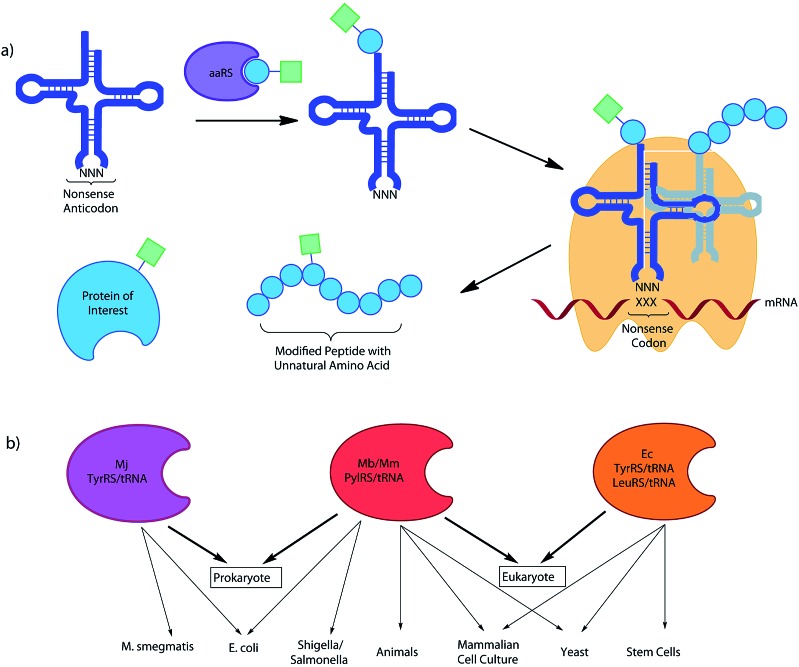
(a) An UAA is charged onto a tRNA with the required non-sense anticodon by an orthogonal aaRS. This tRNA then recognises its corresponding mRNA non-sense codon in the ribosome, leading to incorporation of the UAA into the protein of interest, where it may act as a ‘tag’ for further modification, as a spectroscopic probe, or as a PTM mimic. (b) The organism orthogonality of the 4 most commonly used systems for codon reassignment (*M. jann* TyrRS/tRNA, *M. bar* and *M. maz* PylRS/tRNA and *E. coli* Tyr/LeuRS/tRNA).

Research in the field of codon reassignment has mostly focussed on the application of four main aaRS/tRNA pairs, the TyrRS/tRNA of *M. jannaschii* (*Mj-Tyr*), the *E. coli* TyrRS and LeuRS/tRNA pairs (*Ec-Leu*, *Ec-Tyr*), and the pyrrolysine aaRS/tRNA pair of methanogenic archaebacteria (*Mb-* or *Mm-Pyl*). While overlap certainly exists, in general each provides complementary UAA selectivity in different organisms (Fig. 2). Each will be discussed in turn, with a brief description of their identification and development as a non-sense-suppressor, followed by a focus on the varied UAA structures that have been incorporated.

### TyrosylRS

#### *M. jannaschii* TyrRS/tRNA pair

The TyrRS/tRNA pair from the archaebacteria *Methanococcus jannaschii* was first identified as a functioning suppressor pair in *E. coli* by Wang *et al.*,^34^ before later being utilized for the first incorporation of a UAA, *p*-methoxyphenylalanine (**1**), by amber-stop codon suppression.^7*a*^ The successful application of this pair relies upon the absence of a major anticodon binding region in the aaRS, along with a significantly altered specificity between the *M. jann* and *E. coli* tRNA acceptor loop to generate orthogonality.^35^ The development of an efficient +ve/–ve selection technique, based on the suppression of a chloramphenicol and barnase gene respectively, has allowed the rapid screening and incorporation of a wide range of functionalized aromatic UAAs, most of which are functionalised at a γ-phenyl ring.^36^

Many early examples of incorporated UAAs contained reactive groups for bioconjugation at the *para*-position of the phenyl ring for undertaking subsequent chemical protein modification. For example, reactive allyl (**2**),^36,37^ propargyl (**11**)^38^ and alkynyl groups (**41**)^39^ have been incorporated to install unsaturated carbon–carbon bonds into proteins, while aryl halide (**44** and **45**),^40^ azide (**6**),^41^ aniline (**7**),^36,40a^ ketone (**10**),^42^ diketone (**16**)^43^ and boronic acid (**26**)^44^ functionalities have all been used to provide reactive handles for further modification.^45^ The incorporation of the aniline based *p*-aminophenylalanine (**7**) is particularly noteworthy as it has been demonstrated that the amino acid can be biosynthesised in *E. coli* by hijacking the cellular machinery for the synthesis of aromatic amino acids, effectively generating a bacterium with a 21 amino acid genetic code.^46^ More recently, reactive handles for undertaking the inverse-electron demand Diels–Alder (tetrazine, **54**),^47^ ‘photo-click’ (tetrazole, **55**)^48^ and azo coupling (2-naphthol, **134**)^49^ reactions have been incorporated using this pair. Photocrosslinking amino acids have also been extensively used to probe protein–protein interactions and ligand binding. Benzophenone **5** has been most widely incorporated due to its chemical stability,^50^ though arylazide (**6**)^41^ and diazirine (**20**)^51^ UAAs have also been incorporated.

A number of *para*-substituted-Phe UAAs containing functional probes that do not require further modification for functionality have also been incorporated. For example, fluorescence quenching nitro (**12**),^52^ IR-^53^ and FRET-^54^ active cyano (**17**), NMR-active trifluoromethane (**42**)^55^ and photoisomerizable azobenzene (**14**)^56^ groups have all been incorporated. However, the use of the *M. jann* pair has not only been used to incorporate novel functionalities. The natural post-translationally modified AA sulfotyrosine (**13**) has been installed to study native sulfated proteins,^21^ while it has been suggested that *p*-carboxymethylphenylalanine (**19**) can act as a mimic of the natural modification phosphotyrosine.^57^

While most common, the substrate specificity of the *M. jann* pair does not have to be limited to *para*-substituted phenylalanine derivatives. Through suitable mutation of the TyrRS binding pocket a wider variety of aromatic substituents can be tolerated. For example, expansion of the pocket allows a number of *meta*-substituted tyrosine derivatives to be incorporated, including 3-aminotyrosine (**18**),^58^ used extensively as a probe of radical propagation;^59^ 3-nitrotyrosine (**36**),^23^ a naturally occurring marker of photo-damage; 3,4-dihydroxyphenylalanine (**39**),^60^ a redox-active UAA and 3-iodotyrosine (**4**).^61^ *Meta*-acetyl (**51**)^62^ and *ortho*-nitro (**33**) substituted phenylalanines have also been incorporated, the latter allowing the photo-induced cleavage of a protein backbone.^63^

The binding pocket has also been disrupted to allow the incorporation of alternative aromatic rings, including heteroaromatics. Fluorescent naphthyl (**3**),^40a,64^ hydroxyl (**53**)^65^ and methyl (**52**)^65*a*^-coumarins and hydroxylquinolines (**24**)^66^ can all be used as probes of unfolding and protein structure. Hydroxylquinoline **24** may alternatively act as an efficient chelator of zinc^2+^ ions,^66^ while copper^2+^ ions have been shown to bind to a bipyridine based UAA **22**.^67^ The structure of the coumarin UAAs **52** and **53** are particularly intriguing, as unlike almost all other structures incorporated by the *M. jann* TyrRS/tRNA_CUA_ pair, the coumarinyl-UAAs contain an additional methylene group leading to a δ-, rather than γ-linked aromatic. This is a structure observed in only one other UAA incorporated *via* such a pair: phenylselenocysteine (**48**), although this contains a bulkier selenium atom at the γ-position which may go some way towards fulfilling the steric bulk required by the aaRS.^68^

Phenylselenocysteine is a member of another class of UAAs that can be incorporated utilising a *M. jann* pair, those that are used as a ‘protected’ or ‘latent’ precursor to an amino acid of interest. Phenylselenocysteine itself can be oxidatively eliminated to give the α,β-unsaturated amino acid dehydroalanine (Dha, a natural PTM in some peptide natural products, as well as a reactive handle for further chemical modification).^68,69^ ‘Protected’ tyrosine residues can also be installed, allowing for the selective activation of a protein under the requisite deprotection conditions. *p*-Boronophenylalanine (**26**) can be converted to either tyrosine or phenylalanine by oxidation or reduction respectively,^44^ and has thus been used as a fluorescent peroxynitrite sensor.^70^ While *O*-nitrobenzyltyrosine (**43**) can also be activated under photo de-caging conditions,^71^ a similar de-caging was used to generate fluorinated tyrosine analogues (**46** and **47**), important isosteric probes of tyrosine p*K*_a_ and activity, that cannot be incorporated directly due to their cross-reactivity with the natural cell machinery.^72^

**Fig. 3:**
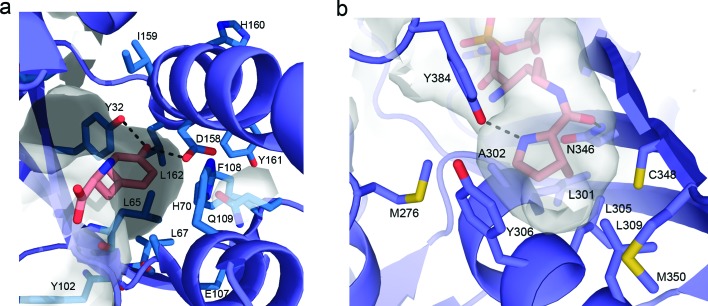
(a) Representation of the active site of the wt *M. jann* TyrRS (taken from PDB file ; 1j1u). Commonly mutated residues are drawn as sticks and labelled. Direct hydrogen bonding between the Tyrosine phenol and Y32 and D168 are indicated. (b) Active site of the wt *M. mazei* PylRS (taken from ; 2q7h). Hydrogen bonding between the side chain carbonyl oxygen of pyrrolysine and N346 and between the pyrroline nitrogen and Y384 are indicated.

Initial efforts to rationally design *M*. *jann* TyrRS substrate specificities were based on the crystal structure of the homologous, but distinct, TyrRS of *Bacillus stearothermophilus*.^73^ Solving the crystal structure of the *M. jann* TyrRS allowed for an increased ability to screen for mutant synthetases and amino acid specificities (Fig. 3a). An observed high degree of structural plasticity goes some way to explaining the adaptability of this system.^74^ This crystal structure has even allowed the design of a pair for the introduction of an ester bond into protein backbones *via* an α-hydroxy acid **35**. This was enabled through mutation around the amino binding region of the aaRS, although significant disruption of the cellular machinery was required to prevent metabolisation of the substrate.^75^

#### *E. coli* TyrRS/tRNA pair

While the *M. jann* TyrRS/tRNA pair is orthogonal in *E. coli*, this is not the case in higher organisms. This spurred an interest in the development of novel aaRS/tRNA pairs for codon reassignment, along with a desire to expand the diversity of structures that can be incorporated. To this extent, Edwards and Schimmel first showed that the *E. coli* TyrRS/tRNA pair was orthogonal to the host cell machinery of the model yeast *Saccharomyces cerevisiae*.^76^ The incorporation of a number of tyrosyl-based UAAs with this pair was first demonstrated by Chin *et al.* in *S. cerevisiae*.^7*b*^ However, this was not the first example of amber-stop codon suppression in eukaryotes, Sakamoto *et al.* having previously demonstrated the incorporation of 3-iodotyrosine (**4**) into mammalian Chinese hamster ovary (CHO) cells, albeit with a low fidelity of incorporation.^9*a*^ However, since the *E. coli* tRNA lacks the promoter sequences required for expression in higher eukaryotes, the tyrosyl-tRNA of *Bacillus stearothermophilus* (*Bs*) was utilised since it already contains the internal A- and B-box promoters required for efficient mammalian expression. As such, the *E. coli* TyrRS has been used in two different suppression systems (*E. coli* TyrRS/*Bs*-tRNA and *E. coli* TyrRS/tRNA) which will be discussed in this section in turn.

#### *E. coli* TyrRS/*Bs*-tRNA

The first incorporation of 3-iodotyrosine (**4**) into mammalian cells with an *E. coli* TyrRS/*Bs*-tRNA pair was hindered by competitive tyrosine incorporation.^9*a*^ Significant improvements in fidelity have subsequently been achieved by the addition of a phenylalanine-specific editing domain to the TyrRS.^77^ The groups of Yokoyama^9a,78^ and Schultz^79^ have since gone on to incorporate a number of UAAs (previously incorporated into *E. coli* using the *M. jann* TyrRS pair) into mammalian systems. These include benzophenone (**5**),^78*b*^ ketone (**10**),^79^ iodide (**45**)^79^ and azide (**6**)^79^ containing phenylalanine derivatives, as well as *O*-propargyltyrosine (**11**).^79^ Such UAAs have found particular use in the study of G-protein coupled receptors (GPCRs), amongst the most important signalling proteins in eukaryotic cells.^80^

#### *E. coli* TyrRS/tRNA

Chin *et al.*'s report on the use of an *E. coli* TyrRS/tRNA_CUA_ pair for amber-stop codon suppression in yeast cells detailed the incorporation of methoxy (**1**), iodo (**45**), azido (**6**), acetyl (**10**) and benzoyl (**5**) phenylalanine residues.^7*b*^ This was subsequently followed by the incorporation of *O*-propargyltyrosine (**11**) as a reactive handle for undertaking CuAAC.^81^ Through modification of the promoter sequences associated with the tRNA gene it is possible to greatly improve the incorporation efficiency of these amino acids in yeast,^82^ while the installation of a type-3 Pol III promoter has allowed this pair to be transferred into more challenging mammalian cell lines such as neuronal stem cells.^9b,83^ While primarily used for the incorporation of UAAs in eukaryotic systems, a niche application in *E. coli* by Iraha *et al.* should also be noted. Replacement of the native *E. coli* TyrRS/tRNA pair with the wild type *M. jann* TyrRS/tRNA, followed by the addition of the mutant *E. coli* TyrRS/tRNA_CUA_ pair, allowed the incorporation of 3-iodo (**4**) and 3-azidotyrosine (**75**) into *E. coli* cells in response to the amber-stop codon.^84^

### LeucylRS

#### *E. coli* LeuRS

Like the TyrRS/tRNA pair, the *E. coli* LeuRS/tRNA pair fulfils many of the requirements for use in higher organisms. It has been shown to be orthogonal to all yeast aaRS and tRNAs,^85^ recognition of the tRNA by the LeuRS is not dependent on the anti-codon loop^86^ and it has a large active site lined only by amino acid side-chains,^87^ offering benefits for engineering specificity. Thus, this pair was adapted by Wu *et al.* ^88^ to incorporate α-aminocaprylic acid (**61**), *O*-methyl tyrosine (**1**) and *O*-nitrobenzyl cysteine (**15**). The structural diversity between these 3 UAAs amply demonstrates the flexibility of the *E. coli* LeuRS/tRNA mutant specificity. *O*-Nitrobenzyl cysteine (**15**) constitutes a photocaged cysteine, which can be released by irradiation with UV light. This system has been used to regulate caspase-3 activity, by substituting an active site cysteine with the photocaged variant. Similarly, photocaged 4,5-dimethoxy-2-nitrobenzylserine (**59**) can be incorporated using this pair^85^ and has been used to monitor the kinetics of nuclear transport of Pho4 upon serine de-caging.

Since the initial demonstrations of this pair for UAA incorporation, a variety of structurally diverse amino acids have been introduced. The fluorescent amino acid dansylalanine (**58**) has been incorporated in *S. cerevisiae* and also in human neural stem cells^9*b*^ to act as a moderately effective environmental probe of unfolding.^89^ An improvement in sensitivity can be achieved utilising the prodan-based UAA Anap **73**, incorporated using a LeuRS/tRNA_CUA_ pair mutant generated *via* a two-step strategy.^90^ First, a LeuRS mutant specific for 3-(naphthalen-2-ylamino)-2-aminopropanoic acid (**74**), an analogue of Anap lacking the ketone functionality, was selected for. A second mutant library was then constructed, yielding an Anap-specific LeuRS. This strategy of developing selectivity in steps allows gradual evolution to a desired aaRS *via* structurally similar derivatives, and has been used on a number of occasions when initial aaRS screening has failed to generate suitable mutants for an UAA of choice. In a further demonstration of LeuRS promiscuity, a ferrocene-containing UAA **49** has been incorporated with this pair, the first metallo-amino acid to be introduced into proteins biosynthetically.^91^

Often the structural space that can be incorporated with a single mutant aaRS is quite limited. The generation of promiscuous aaRSs, which can accept multiple different substrates greatly simplifies the introduction of new amino acids by negating the need for aaRS mutation. The LeuRS appears to be well suited to such applications given its tolerance for structural diversity. A promiscuous LeuRS mutant has been shown to be capable of charging different length methionine, cysteine and alkyl analogues, allowing the incorporation of a variety of structures using a single mutant (**61–68**).^92^ Similarly, through mutation of the active and the CP1 editing site of the aaRS, a number of alkene containing amino acids (**60** and **69–72**), of varied length and heteroatom substitution have been incorporated.^93^ Such alkene moieties could perhaps subsequently be used as selective ‘tags’ for chemical modification by olefin metathesis or thiol–ene reactions.

### PyrrolysylRS

#### *Methanosarcina barkeri* and *Methanosarcina mazei* RS/tRNA pair: wild type

The discovery of the ‘22^nd^ amino acid’ (the 21^st^ being selenocysteine) in the genetic code was described in two back-to-back reports in 2002.^94^ Researchers were set onto its trail, by the observation that all methylamine methyltransferases in methanogenic archaea contained an in-frame TAG stop codon, which appeared to be ‘read-through’. The crystal structure of the *Methanosarcina barkeri* monomethylamine methyltransferase then allowed the structure of this novel amino acid to be deduced, revealing a 4-methyl-pyrroline-5-carboxylate, linked to the lysine *N*-ε *via* an amide bond, adequately christened pyrrolysine. The amino acid was inserted co-translationally in response to the amber stop codon, an example of natural suppression. These studies also identified a putative pyrrolysine-selective tRNA_CUA_ and aminoacyl-transferase (PylRS) pair. Further studies revealed that pyrrolysine is charged to its target tRNA as an intact amino acid,^32^ in contrast to selenocysteine, which is synthesised after conjugation of serine to the requisite tRNA. Importantly, it has been shown that PylRS/tRNA_CUA_ pairs are orthogonal in *E. coli*, paving the way for the use of this system for codon reassignment. Indeed, since the aaRS/tRNA pair is derived from an archaebacteria, it has been found that this pair is also orthogonal in eukaryotes, allowing the incorporation of UAAs in yeast and mammalian cell lines, as well as more recently in whole organisms (see Fig. 2b).^47^

Early studies of the PylRS/tRNA pair utilized pyrrolysine mimics to map the biochemical activity of the aaRS, determining the structural features of the amino acid required for recognition. Additional motivation was provided not only by the potential biotechnological applications but by the difficulty in obtaining the amino acid pyrrolysine (necessary for further characterization of the PylRS mechanism but requiring a 16-step synthesis).^95^ Initial analogue studies showed that the PylRS/tRNA_CUA_ pair was able to efficiently incorporate a variety of amide or carbamate substituted lysines, such as 2-amino-6-((*R*)-tetrahydrofuran-2-carboxamido)hexanoic acid (2Thf-lys, **84**),^96^ *N*-ε-d-prolyl-l-lysine (**81**)^95^ and *N*-ε-cyclopentyloxycarbonyl-l-lysine (cyc, **82**).^95^ It was also established that pyrrolysine incorporation was not mediated *via* a distinct mRNA structure,^95^ again in contrast to selenocysteine. The availability of the commercial pyrrolysine analogue cyc (**82**), enabled the structural characterization of a ternary PylRS–AMP-cyc complex (Fig. 3b).^97^ With this structural information in hand, directed evolution and rational design approaches to engineer PylRS specificity have became possible (see the following section for details).

The wild type PylRS has proven remarkably flexible in terms of substrate recognition, allowing the introduction of many useful functional groups, without the need for the generation of mutant synthetases. Typically, the unnatural amino acids present a specific functionality conjugated *via* a carbamate linker to the *N*-ε of lysine. The first examples of chemical handles/‘tags’ for bioconjugation labelling reactions were the introduction of alkyne (**86**) and azide (**90**) functionalities for CuAAC by Nguyen *et al.* ^98^ Similarly, a Boc-protected lysine (**77**) was also incorporated, while Fekner *et al.* ^99^ introduced an amide linked tetrahydrofuran moiety (**85**), which may mimic the pyrrolysine ring, bearing an alkyne to site-specifically label calmodulin (CaM). Selective modification of the pyrrolysine analogue by CuAAC and alkylation of a nearby cysteine with a FRET active dye pair, allowed the tracking of conformational changes in CaM by FRET measurements. The same group also reported the efficient incorporation of another alkyne-containing pyrrolysine analogue *N* ^6^-(2-(*R*)-propargylglycyl)-lysine (**105**),^100^ which was used for the same purpose. In a follow-up, the authors replaced the lysine *N*-ε with an oxygen, substituting the ε-amide for a cleavable ester bond (**106**).^101^

In order to allow expansion of thioester-to-amide (so-called ‘native chemical ligation’, NCL) chemistry, cysteine (both d and l, **92**) or a thiazolidine protected cysteine (**104**) conjugated to the lysine *N*-ε have been incorporated by amber suppression^102^ and used as chemical handles for protein ubiquitinylation *via* NCL,^103^ or fast ‘bioorthogonal’ labelling with 2-cyanobenzothiazoles.^104^ Although these amino acids are incorporated by the wt PylRS, the efficiency is relatively low and can be improved with mutant aaRSs.^104^ In order to allow IEDDA reactions for rapid protein labelling, a carbamate linked norbornene moiety (**101** and **122**) has recently been genetically-incorporated using both wt and mutant PylRSs.^105^ This has allowed rapid modification of target proteins with a tetrazine reaction partner, or by a [3 + 2]-dipolar cycloaddition with nitrile imines. This useful moiety has also been encoded in *Drosophila* cells.^10*a*^ Small aliphatic diazirines (**96**), can also be incorporated using the wt PylRS/tRNA pair.^106^ This photocrosslinker was shown to covalently crosslink glutathione *S*-transferase (GST) dimers both *in vivo* and *in vitro*.

The PylRS/tRNA pair has also been used for the incorporation of naturally occurring PTMs. Mono- and di-methyl-lysine have been incorporated *via* intermediate Boc-protected lysine derivatives (**97** and **77** respectively) and subsequent chemical modification.^107^ This has been particularly useful for the study of histones, which are commonly modified at numerous lysine residues.

A detailed examination of the PylRS crystal structure reveals no significant interaction occurs with the lysine α-amine. This has been exploited by Kobayashi *et al.* to introduce the α-hydroxy acid of Boc-lysine **110** into proteins.^108^ This site-specific substitution of an amide for an ester bond in the protein backbone allows protein cleavage at this site by treatment with ammonium hydroxide (as previously also demonstrated with the *M*. *jann* incorporated hydroxyl acid **35** (ref. 75)).

The use of structural analogues has allowed the deduction of the necessary structural features required for efficient charging of an UAA to the aaRS. The most important is the presence of an amide or carbamate linkage at the *N*-ε. Here, both the planarity of this linkage and the hydrogen bonding capacity of the carbonyl oxygen appear important for recognition. In addition, the cyclic imine provides a further hydrogen bonding motif, which is recognized by the PylRS (Fig. 3b). While not essential, the presence of this hydrogen bond appears to significantly increase tRNA charging by the PylRS, as exemplified by the fact that 2-amino-6-((*R*)-tetrahydrofuran-2-carboxyamido)hexanoic acid (2-Thf-Lys, **84**), which contains an oxygen in the same position as the imine nitrogen in pyrrolysine, is accepted as a substrate by PylRS, but 3- and 4-Thf-Lys variants with differently positioned endocyclic heteroatoms are not. Since the size of the active site is limiting in terms of the potential utility of the wt PylRS/tRNA pair, a number of groups have undertaken directed evolution or rational design to give mutants with altered specificity. Such mutants will be discussed in the next section.

#### *Methanosarcina barkeri* and *Methanosarcina mazei* mutant PylRS/tRNA pairs

Although the wild type PylRS shows good substrate flexibility towards a number of pyrrolysine analogues, the advantages of the PylRS/tRNA pair in terms of species orthogonality encouraged the evolution of the synthetase towards more diverse structures. As a result, a number of mutant PylRS/tRNA pairs with significantly broadened substrate scope have been reported, facilitating the incorporation of UAAs with bulky groups at the *N*-ε atom of l-lysine. Importantly, the orthogonality of the pair in both prokaryotic and eukaryotic cells enables the evolution of the enzyme in a fast-growing system such as *E. coli*, followed by direct transfer of the mutant to eukaryotic cells where evolution is more challenging.

In general, the mutants may be designed either rationally or by randomization of a number of active site residues of the PylRS. The randomized residues are either selected arbitrarily, or on the basis of the crystal structure of the *M. mazei* (*Mm*) PylRS in complex with pyrrolysine (PDB entry ; 2q7h).^97,109^ Since the binding pocket residues are well-conserved between the *Mm*-PylRS and *M. barkeri* (*Mb*)-PylRS,^110^ this model has been used for the engineering of both enzymes. The analogous *Mb*-PylRS and *Mm*-PylRS mutants indeed show similar efficiencies for the incorporation of the same substrate.^111^ As such, the two systems are often used arbitrarily based on the system available to the interested research group, and can in effect be used interchangeably.

The first example of a PylRS mutant for codon reassignment was reported by Chin and co-workers in 2008, when an *Mb*-PylRS mutant was evolved to incorporate the extensively studied natural lysine PTM *N*-ε-acetyllysine (**78**) in *E. coli*. The mutant *Mb*-PylRS possessed 6 mutations thought to partially plug the large hydrophobic cavity present around the pyrroline ring of the wt synthetase, while still accommodating the acetyl group.^110^ The same mutant was later shown by Liu and co-workers to successfully incorporate the corresponding alkyl analogue 2-amino-8-oxononanoic acid (**118**) in *E. coli*.^112^ Shortly after, Yokoyama and co-workers engineered a *Mm*-PylRS bearing 3 mutations at random sites and two in the pyrrolysine binding pocket, creating sufficient room to accommodate the large benzyloxy group of *N*-ε-benzyloxycarbonyl-l-lysine (**76**) and enable its incorporation into recombinant proteins in mammalian cells.^111^

Yokoyama and co-workers have shown that a single mutation of *Mm*-PylRS (Y384F), identified by random screening, significantly improves the amber suppression efficiency for BocLys (**77**) and AllocLys (**107**) compared to the wild type synthetase in *E. coli*. A second mutant, containing the structure-based mutation Y306A, in addition to Y384F, provided good yields for the incorporation of *N*-ε-benzyloxycarbonyl-l-lysine (**76**) and also enabled the large scale incorporation of *N*-ε-(*O*-azidobenzyloxycarbonyl)-l-lysine (**80**) in *E. coli*.^113^ The same *Mm*-PylRS mutant has been used for the incorporation of a variety of UAAs in *E. coli* including *N*-ε-[2-(furan-2-yl)ethoxy]carbonyl-lysine (**140**) for photo-activated protein–RNA cross-linking^114^ and Se-alkylselenocysteines (**89**).^115^ In addition, a number of tags designed for selective reactions have been incorporated, such as various norbornene (**101** and **122**),^116^ transcyclooctene (**100**)^116,117^ and cyclooctyne (**99** and **102**) derivatives^118^ in *E. coli* and mammalian cells. Small variations of this mutant, have enabled the incorporation of the two photo-crosslinking UAAs *N*-ε-(*p*-(nitrobenzyloxycarbonyl))-l-lysine (**91**) and *N*-ε-[((4-(3-(trifluoromethyl)-3*H*-diazirin-3-yl)-benzyl)oxy)carbonyl]-l-lysine (**117**) in *E. coli* and mammalian cells.^119^ Chin and co-workers have also demonstrated the incorporation of other selective reaction handles such as transcyclooctenes (**100**) and cyclooctynes (**99**) derivatives with *Mb*-PylRS mutants in *E. coli* and mammalian cells, for undertaking strain-promoted azide–alkyne cycloadditions and inverse-electron demand Diels–Alder reactions.^120^ A similar mutant was used for the incorporation of a five-membered 2,2,5,5-tetramethyl-pyrrolin-1-oxyl spin label moiety **139**.^121^ The incorporation of the photocaged lysine derivative *O*-nitrobenzyl-oxycarbonyl-*N*-ε-l-lysine (**91**) was demonstrated by Schultz and co-workers with a *Mm*-Pyl mutant in *E. coli* and mammalian cells.^122^ Liu and co-workers later reported the encoding of a photocaged mono-methyllysine analogue **98** in *E. coli*. Irradiation of proteins containing this amino acid results in site-specifically incorporated methyl-lysine, for which no selective aaRS has been evolved to date.^123^ *N*-ε-Acryloyl-l-lysine (**115**) has also been encoded and shown to undergo conjugation reactions in *E. coli*.^124^ This amino acid was also used to undertake ‘photo-click’ chemistry in *E. coli*, mammalian cells and even plants (*A. thaliana*).^125^ More recently, spiro-hexene **115** has been incorporated using the same pair to improve the rates of ‘photo-click’ reactions.^126^

Chin and co-workers have extended the technology further by engineering a PylRS/tRNA_CUA_ pair functional and orthogonal in yeast. Different *Mb*-PylRS mutants have been evolved for the incorporation of *N*-ε-acetyl-l-lysine (**78**), and its analogues trifluoroacetyl-l-lysine (**93**) and photocaged lysine derivative *N*-ε-[(1-(6-nitrobenzo[*d*][1,3]dioxol-5-yl)ethoxy)carbonyl]-l-lysine (**94**).^127^ The same group subsequently demonstrated the incorporation of photocaged-lysine **94** in mammalian cells with an *Mb*-PylRS mutant.^128^ A similar mutant enabled the incorporation of the photoreactive *N*-ε-(1-methylcycloprop-2-enecarboxamido)lysine (**103**) in *E. coli* and mammalian cells.^129^ The group also demonstrated the incorporation of a photocaged cysteine analogue **141** in *E. coli* and mammalian cells.^130^

Chen and co-workers originally identified an *Mb*-PylRS mutant for the incorporation of the reactive azide *N*-ε-(((1*R*,2*R*)-2-azidocyclopentyloxy)carbonyl)-l-lysine (**87**) in *E. coli* and mammalian cells.^131^ This mutant has subsequently been used for the incorporation of a variety of UAAs in *E. coli* such as α-hydroxy-BocLys (**110**)^132^ or chemical handles such as alkenylpyrrolysine (**107–109**)^133^ and alkynylpyrrolysine (**86**, **112** and **113**) analogues.^134^ A related mutant was identified for the incorporation of the photo-crosslinker 3-(3-methyl-3*H*-diazirine-3-yl)-propaminocarbonyl-*N*-ε-l-lysine (**96**) in *E. coli* and mammalian cells^135^ and subsequently for the incorporation of aryl iodide (**114**) and alkynylpyrrolysine (**113**) analogues in the Gram-negative bacterial pathogens *Shigella* and *Salmonella*.^8*a*^ Further single-site mutation enabled the incorporation of the newly identified natural histone PTM *N*-ε-crotonyl-l-lysine (**121**).^136^

One single mutation to the wild type *Mb*-PylRS, C313V enables significant improvement in the incorporation efficiency of the 1,2-aminothiols *N*-ε-d-cysteinyl-l-lysine and *N*-ε-l-cysteinyl-l-lysine (**92**) in *E. coli*.^104^ In the same paper, further evolution of the synthetase enabled the incorporation of *N*-ε-l-thiapropyl-l-lysine (**104**) through three additional mutations.^104^

Remarkably, PylRS can be evolved to incorporate unnatural amino acids bearing aromatic cores instead of the classical pyrrolysine –(CH_2_)_4_–NHR scaffold, while still remaining orthogonal towards endogenous amino acids, demonstrating the flexibility of the aaRS binding pocket towards substrate recognition. In a first example, Liu and co-workers demonstrated that the *Mm*-PylRS/tRNA_CUA_ pair can be evolved to preferentially tolerate l-phenylalanine (**148**), and its derivatives *p*-iodo- (**45**) and *p*-bromo-phenylalanine (**44**), whose side chain structures are drastically different from pyrrolysine.^137^ Strikingly, the same group reported a rationally designed *Mm*-PylRS-(N346A, C348A) mutant able to efficiently incorporate seven different *O*-substituted tyrosine derivatives (**1**, **2**, **11**, **31**, **50** and **167**)^138^ and twelve different *meta*-substituted phenylalanine analogues (**146**, **147** and **157–166**)^139^ bearing diverse chemical functionalities in *E. coli*. This is particularly remarkable since *meta*-substituted phenylalanine derivatives are in general incorporated poorly by previously reported *M. jann* and *E. coli* TyrRS mutants. Notably, the same mutant was able to incorporate seven *ortho*-substituted phenylalanine derivatives (**149–155**)^140^ and thirteen *meta*-alkoxy- and *meta*-acyl-phenylalanines (**135–138**, **156** and **142**),^141^ bringing the number of phenylalanyl based UAAs incorporated by this single mutant to almost 40. Similarly, Arbely *et al.* have reported the incorporation of photocaged *o*-nitrobenzyl-*O*-tyrosine **43** using a *Mb*-PylRS mutant in *E. coli* and mammalian cells.^142^ More recently, Schultz and co-workers reported the incorporation of a number of functionalised histidine analogues (**129–133**), further demonstrating the plasticity of the PylRS,^143^ while Wang's group used a mutant library generated by optimized saturation mutagenesis for the creation of mutants that incorporate conjugated aromatic rings (**5** and **128**).^144^

### Miscellaneous aaRSs

The previously described aaRS/tRNA pairs have proven to be both robust and flexible, as illustrated by the multitude of structures that can be incorporated and the many applications in which they have been used. Interest remains though in the development of additional aaRS/tRNA pairs that are orthogonal in commonly-used model organisms. This could expand the structural space of incorporated amino acids and provide additional systems that can be combined for the incorporation of multiple amino acids (see following section). Towards this goal, a variety of novel pairs from different organisms have been explored. The *Pyrococcus horikoshii* glutamyl^145^ and lysyl,^12^ *S. cerevisiae* aspartyl,^146^ glutaminyl,^30^ tyrosyl^147^ and tryptophanyl,^148^ *Methanobacterium thermoautotrophicum* and *Halobacterium* sp. leucyl,^149^ *Pyrococcus horikoshii* prolyl and *Archaeoglobus fulgidus* prolyl^150^ aaRS/tRNA pairs have all been reported to be orthogonal in *E. coli*. In yeast the *E. coli* glutaminyl,^147*b*^ and in mammalian cells the *Bacillus subtilis* tryptophanyl^151^ aaRS/tRNA pairs have both also been shown to be orthogonal.

UAA incorporation has been demonstrated utilising the *P. horikoshii* LysRS/tRNA pair that has also been used to incorporate homoglutamine (**123**) in response to a quadruplet codon (discussed further below). Meanwhile, the *Methanosarcina acetivorans* TyrRS/tRNA pair has been used to incorporate 3-azidotyrosine (**75**) and 3-iodotyrosine (**4**),^152^ while the *Bacillus stearothermophilus* TrpRS/tRNA pair allows the incorporation 5-hydroxytryptophan (**124**) into the foldon protein in mammalian cells. This amino acid possesses a distinctive absorption band at 310 nm and is also redox active, allowing for the oxidative cross-linking of a foldon protein dimer when a positive potential is applied. In *E. coli* a polyspecific *S. cerevisiae* yeast TrpRS/tRNA pair has also been used to incorporate a variety of tryptophan analogues (**124–128**) to replace the central tryptophan in the ECFP chromophore.^148*b*^ This has a pronounced effect on the fluorescent properties of the protein, dependent on the choice of UAA. Also of particular note is the use of the *M. jann* Cys-tRNA and *Methanococcus maripaludis* phosphoserine-RS pair for the incorporation of the natural posttranslational modification phosphoserine (**167**).^22^

Many of the above mentioned pairs have not passed past the proof-of-orthogonality stage and have not yet been used to incorporate UAAs. Causes for this are certainly specific for each individual pair, but a lack of evolvability or inefficient suppression are commonly-encountered shortcomings. As such, the development of novel orthogonal aaRS/tRNA pairs still remains a relevant challenge.

### Systems for incorporation of multiple amino acids

**Fig. 4:**
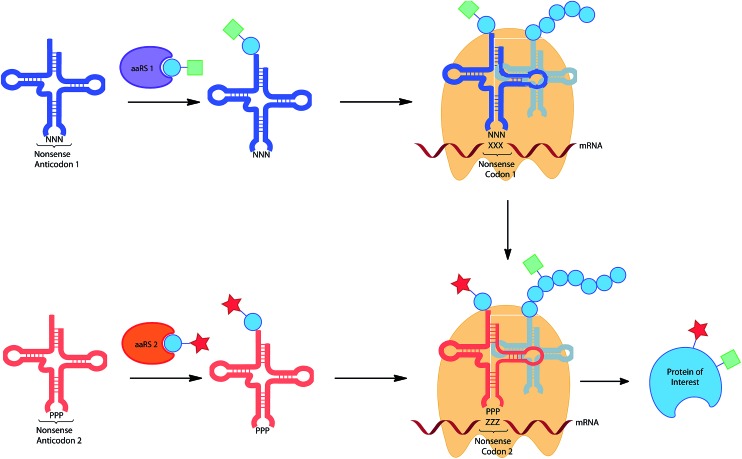
Incorporation of multiple UAAs into the same protein utilising the reassignment of 2 different non-sense codons.

The wealth of UAAs that can be incorporated by codon reassignment already provides an important toolbox for biochemical studies of protein function. However, for more sophisticated purposes the incorporation of multiple functionalities into proteins would be desirable. For example, the incorporation of two different ‘tags’ for chemical modification would allow the addition of two complementary dyes for FRET measurements. Similarly, the incorporation of a photocrosslinker and a modifiable moiety, could allow highly specific capture and release, desirable for proteomic approaches to protein study. The *in vivo* incorporation of multiple unique amino acids is being pursued by a number of groups, currently only by two general approaches: the use of two distinct stop codons and the use of a stop codon in combination with a quadruplet codon (see below and Fig. 4).

The first approach was initially realized by combining a *para*-azidophenylalanine specific *M. jann* TyrRS/tRNA_CUA_ and the wild type PylRS/tRNA pair.^11^ The authors first investigated whether the mRNA codon recognized by the Pyl tRNA could be changed from the naturally recognized UAG stop codon to UAA (ochre), UGA (opal) or a quadruplet UAGA codon. While the quadruplet codon significantly decreased protein yield, UAA and UGA actually resulted in higher protein expression when compared to the amber codon. An ochre specific PylRS/tRNA_UUA_ pair was therefore combined with a *M. jann* TyrRS/tRNA_CUA_ pair to incorporate both azide (**6**) and alkyne (**86**) tags into GFP. The two orthogonal moieties were individually labelled with two different fluorescent dyes *via* a CuAAC reaction, resulting in the formation of a FRET pair. In a later report it was shown that by choosing appropriate conjugation chemistries, in this case strain-promoted azide–alkyne cycloaddition and hydrazone forming reactions, two different labels could be conjugated to a single protein in a one pot procedure.^33*b*^ Subsequent detailed analysis of suppression efficiency of all three stop codons by different aaRS/tRNA variants, will allow further expansion and optimization of this approach.^153^ While optimization of the aaRS/tRNA-bearing plasmid can greatly improve expression yields for the incorporation of a single UAA.^31*c*^ Similarly, Chatterjee *et al.* have presented an optimized system for the incorporation of two distinct UAAs.^33*a*^ The constructed system allows the encoding of both distinct aaRS/tRNA pairs on a single plasmid, termed pUltraII, greatly simplifying incorporation.

## Global suppression

While codon reassignment has the advantage of giving site specific incorporation of the desired UAA, the global replacement of a particular amino acid is another efficient way for the incorporation of single or multiple UAAs and is often sufficient to yield proteins with the desired biophysical property (*e.g.* enhanced stability or fluorescence). Global amino acid replacement commonly utilises auxotrophic strains, incapable of synthesizing a particular amino acid (or multiple amino acids if the strain is polyauxotrophic), to substitute residues with structurally similar counterparts. The most prevalent examples of replacement are the introduction of alkyne^154^ and azide^155^ functionalities through the substitution of methionine (Met) by azidohomoalanine (Aha) or homopropargylglycine (Hpg). These amino acids can then be used as reactive handles for further chemical modification, such as through CuAAC. The introduction of Aha is not limited to incorporation in auxotrophic strains of *E. coli*; in human cell culture Aha can be incorporated into proteins when supplied in the growth medium instead of Met. This incorporation in mammalian cells has been used to identify newly synthesized proteins with great temporal resolution, *via* pulse-chase labelling experiments.^156^ The need to incorporate UAAs that are structurally similar to endogenous amino acids^157^ is a limitation of this technique, yet still the functional space of proteins can be greatly expanded using codon suppression.^158^ The structural restraints can be partially alleviated by using engineered aaRSs that have relaxed specificity towards their natural substrate, increasing the number of UAAs that can be incorporated. A key advantage of global replacement is that multiple incorporations of the same, or up to three different UAAs, can be accomplished in the same protein while retaining good yields.^159^ Interestingly, these replacement experiments have generated proteins consisting of up to 10% of UAAs, which were shown to still retain activity.^159*a*^ Combining UAA incorporation with directed evolution can further help to generate proteins with desired properties such as increased folding rates.^160^ The use of global replacement is in theory compatible with all codon reassignment methods making the combination of different UAA incorporation techniques an attractive future direction for further expanding protein function.

## Quadruplet codon suppression

The number of distinct UAAs that can be encoded by an organism is theoretically limited by the availability of non-coding triplet codons. Utilizing different codons within the same sequence with different register can also be viewed as a good strategy for increasing coding power. As a result, quadruplet codons have been used as an alternative for encoding UAAs. Early reports described the stoichiometric use of pre-aminoacylated, extended quadruplet anticodon tRNAs to incorporate UAAs in response to four-base codons *in vitro* or when microinjected in to *Xenopus* oocytes. These systems however require the synthesis of the pre-aminoacylated tRNA precursor, show low efficiency of incorporation and are not applicable to most cell types or larger scale expressions.^161^

Here, we will focus on quadruplet codon-suppression involving orthogonal synthetase/tRNA pairs, able to selectively aminoacylate the tRNA and decode the quadruplet codons to incorporate UAAs *in vivo*. The first example of such a system was reported by Schultz and co-workers using a variant *Pyrococcus horikoshii* LysRS/tRNA pair in *E. coli* to incorporate l-homoglutamine (**123**) in response to the quadruplet codon AGGA. The orthogonality of this pair to the *M. jann* TyrRS/tRNA_CUA_ pair enabled the simultaneous incorporation of *O*-methyl-l-tyrosine (**1**) into recombinant myoglobin.^12^ However, this system proceeded with relatively low efficiency. Indeed, although the natural ribosome is capable of recognizing quadruplet anti-codon tRNAs, their decoding is relatively inefficient. This can be rationalized by a poor accommodation of the extended tRNA codon-binding region in the ribosome. At the same time, the natural ribosome is not evolved towards more efficient quadruplet decoding since this might lead to misreading and missynthesis of the proteome, toxicity and cell death.

To overcome this problem, further engineering of the cellular translational machinery has been achieved by Chin and co-workers, who have developed an orthogonal translation pathway within the cell. This has involved the creation of an orthogonal ribosome recognizing an alternative Shine–Dalgarno sequence, a short sequence upstream of the 5′-AUG translation initiation codon responsible for mRNA recognition by the ribosome in prokaryotes. This orthogonal ribosome was shown to be selectively directed to corresponding orthogonal mRNAs bearing such a Shine–Dalgarno sequence. The orthogonal mRNA is in turn not recognized by the endogenous ribosome.^162^ This orthogonal ribosome can be evolved towards a quadruplet code by mutating residues around the ribosome A site, responsible for the fidelity of triplet decoding. This ‘loosening’ enables efficient quadruplet codon decoding without affecting the translation of cellular mRNAs by natural ribosomes, and thus without toxic misreading of the proteome.^163^ In the presence of sufficient aminoacylated tRNA, the level of quadruplet decoding can approach that of triplet decoding by the natural ribosome.^163^

This orthogonal translation machinery was first used for quadruplet codon decoding using a system based on the *M. jann* TyrRS/tRNA_CUA_ pair. First, a *M. jann* tRNA_UCCU_ was developed together with a synthetase able to identify *p*-azido-l-phenylalanine (**6**) and to recognize the quadruplet anticodon. Using this *M. jann* AzPheRS/tRNA_UCCU_ variant, *p*-azido-l-phenylalanine (**6**) was incorporated in response to the AGGA codon in *E. coli*. The *M. jann* AzPheRS/tRNA_UCCU_ pair, being orthogonal to the *Mb*-PylRS/tRNA_CUA_ pair, allowed simultaneous incorporation of *p*-azido-l-phenylalanine and an aliphatic alkyne (**86**) *via* quadruplet and amber codon suppression respectively in a single protein.^163^ Further evolving Pyl tRNA for quadruplet codon decoding has enabled optimized translation of quadruplet codons. Chin *et al.* recently reported the evolution of several PylRS/tRNA_XXXX_ pairs, thereby enabling double quadruplet suppression in a single protein. In addition, using *Mb* PylRS/tRNA_UACU_ and *Mj* TyrRS/tRNA_CUA_ pairs, two unique amino acids, norbornyl-lysine (**122**) and tetrazinyl-phenylalanine (**54**) were incorporated in the Ca^2+^-binding protein calmodulin with efficiencies of up to 20%. Elegantly using two orthogonal reactions involving a more activated tetrazine and a bicyclononyne probe, they achieved a one-pot incorporation of a FRET pair on the protein.^164^

In an alternative strategy, Schultz and co-workers proposed an engineered Pyl-tRNA_UCCU_ to incorporate *N*-ε-(*tert*-butyloxy-carbonyl)-l-lysine (Boc-Lys, **77**) in response to the AGGA codon in both bacterial and mammalian cells. The evolution of the tRNA improved aaRS recognition and ribosome affinity, and therefore enhanced the efficiency of quadruplet codon decoding by the natural ribosome, and without need for further engineering of the host cell translational machinery.^165^

Quadruplet decoding opens up interesting possibilities for the incorporation of multiple UAAs into proteins, by providing, theoretically, 256 additional blank codons. However, this approach is limited by the number of available aaRS/tRNA pairs orthogonal to both host cell machineries *and* to each other. Current efforts to further evolve new orthogonal synthetases and tRNAs *de novo* may soon lead to systems able to incorporate more than two different UAAs in a single protein.

## Sense codon suppression

The reassignment of ‘non-sense’ codons has developed into a powerful tool for UAA incorporation, yet is still limited by competition with the host translational termination machinery and resultant low protein yields. As will be further discussed in the following section, one solution to this problem is by preventing termination as described by Johnson *et al.*, deleting the ‘TAG’-specific release factor RF1, thus allowing efficient reassignment.^166^ However, the suppression of ‘native’-TAG codons may then subsequently begin to affect host fitness through the undesirable extension of peptide sequences.^167^ A further solution to this problem has been devised by Isaacs *et al.* Through the development of a technique known as hierarchical conjugative assembly genome engineering (CAGE), all 314 naturally occurring TAG codons in the genome of *E. coli* can be replaced with an alternative stop codon, thus minimising any deleterious effects of codon reassignment.^168^ It was subsequently shown that such engineering allows the deletion of RF1 and the complete reassignment of the TAG codon for UAA incorporation, even in strains for which RF1 knockout is usually lethal.^169^

This ability to create an ‘amber-free’ system by completely replacing a codon throughout a genome highlights the potential of another powerful tool that we have covered only briefly thus far. The utilization of sense codons to incorporate UAAs has a long history^170^ and has been enhanced by the availability of associated auxotrophs to enable greater control (see ‘Global suppression’ section above). This is already an existing powerful form of sense codon utilization. Genome ‘editing’ now additionally opens the door to the reassignment of degenerate ‘sense’ codons. Since most amino acids are translated by multiple codons, those which are rarely used may be ideal candidates for such reassignment. Given that, in theory, up to 20 such codons may be replaced while maintaining a viable genome with no competition from the host termination machinery, such codons offer a potential technique for the efficient incorporation of multiple UAAs.^171^ However, significant challenges remain to be overcome in order to achieve such goals. The scarcity of ‘natural’ reassignments highlights the challenges that may be encountered due to the possibility of differing translational efficiencies associated with seemingly equal codons, or hidden effects on gene regulation. Indeed, in pioneering work by Lajoie *et al.* the removal of 13 rare codons from a number of essential genes proved to be widely tolerated by the host, yet a decrease in fitness in many instances demonstrated that ‘synonymous codons can be non-equivalent in unpredictable ways’.^172^

Another challenge is the misidentification of UAA-associated tRNAs by host synthetases. Krishnakumar *et al.* have shown that while the Pyl-tRNA_CUA_ is efficiently aminoacylated by the Pyl-aaRS, when suppression of the arginine codon CGG was attempted, using the altered anti-codon Pyl-tRNA_CCG_, unexpected misacylation with arginine was observed as the major product.^171^ This can be attributed to the use of the tRNA-anticodon as a major recognition element for many aaRSs, leading to a need for further engineering than simply native-tRNA knockout for ‘sense’-codon reassignment. There may therefore be a need to focus on codons which do not rely on such recognition, or alternatively the addition of anti-determinants of recognition to minimise misacylation.^173^

Despite these potential difficulties, important early steps towards ‘sense’-codon reassignment have already been achieved by a number of groups. Bohlke and Budisa have shown that the rare isoleucine-codon AUA can be liberated from its natural translation pathway *via* tRNA knockout and subsequent replacement with a tRNA from an alternative species, albeit continuing to incorporate isoleucine.^174^ More recently, Bröcker *et al.* have demonstrated that the incorporation machinery of selenocysteine (the rarely used ‘21^st^ amino acid’, see reviews by Böck *et al.* ^175^ and Johansson *et al.* ^176^ for details) can be modulated to recognise 58 of the 64 naturally occurring codons, in many cases completely outcompeting the endogenous tRNA, although other codons resulted in ambiguous translation.^177^ These reports demonstrate that while limitations still exist and UAAs are yet to be incorporated, ‘sense’-reassignment remains an exciting topic of research. In particular, while many rare codons or tRNA/aaRS systems may prove not to be of use, further research into the subtleties of tRNA recognition and the interactions of tRNA/aaRS with the host machineries may prove highly fruitful in allowing UAA incorporation with greatly improved efficiency.

## Outlook

Enormous strides have been made in the field of codon-reassignment in little over a decade, yet there remain a number of key challenges to be overcome. The vast majority of studies so far have focused on the incorporation of UAAs in single cell cultures. While this has proved a powerful tool for understanding a number of important cellular processes, a transition into multicellular organisms would be an important discovery for studying inter-cellular functions. To this extent a number of recent studies have begun to address the challenges of genetic code expansion in animals.

Chin and co-workers, first demonstrated the incorporation of *N*-ε-(*tert*-butyloxy-carbonyl)-l-lysine (BocLys, **77**) and *N*-ε-[(2-propynloxy)carbonyl]-l-lysine (**85**) in *Caenorhabditis elegans* using a wt *Mm*-PylRS/tRNA_CUA_ pair.^10*b*^ In order to generate a worm containing the required genetic information, they used ‘biolistic’ bombardment to deliver an extrachromosomal array, requiring antibiotic-based selections to maintain the DNA constructs in the cell line. While undoubtedly an important discovery, suppression efficiencies were very low and observed only in a small subset of transformed worms. A similar technique was used for the expansion of the genetic code of *Drosophila melongaster*. Suppressions were undertaken in both fly embryos, and in specific tissues and cell subsets in adult flies.^10*a*^

In an alternative approach, Parrish *et al.* reported the dual encoding of *O*-methyltyrosine (**1**) and dansylalanine (**58**) with *E. coli* TyrRS/tRNA and LeuRS/tRNA pairs respectively, again in *C. elegans*. In this case however, they first generated stable transgenic worms with chromosomally integrated reporter and aaRS/tRNA genes, resulting in increased levels of suppression and genetic stability.^178^ Further optimisation of incorporation may have important implications in the study of embryonic development, neural processing and cancer biology that can only be addressed in an *in vivo* setting. Recently, Wang and co-workers transferred this technology into plants, using a MbPylRS mutant incorporating an acryllysine UAA **111** as a handle for ‘photo-click’ chemistry, into proteins. This was achieved by first constructing a vector containing the corresponding genes that could replicate in *Agrobacterium*, which could in turn deliver the genes into the plant host.^125^

Another significant challenge in the field is the improvement of suppression efficiency. Current non-sense techniques suffer from UAA incorporation, in effect, being in direct competition with protein truncation. As such, incorporation efficiency can be drastically reduced and in many cases fails entirely. Knock out of RF1, the release factor responsible for termination at UAG, eliminates the problem of competing termination.^166^ Since this protein is primarily responsible for decoding the amber stop codon, UAG, and causing translational termination, its deletion reduces competition and greatly increases incorporation efficiency. As a result, up to 10 stop codons can be suppressed in a single protein. Similar discoveries are now required in eukaryotes in order to improve the efficiency of UAA incorporation in these more challenging cell lines. An efficient codon reassignment will also likely greatly aid the incorporation of multiple amino acids into the same protein (see above) using multiple orthogonal RS/tRNA pairs.

While over 150 UAAs have been incorporated by codon reassignment, these are mainly based around the same few core structural motifs. There are many structures or functionalities that still cannot be incorporated, and as such there remains a need to discover additional orthogonal pairs that can be used. In particular, there are many natural PTMs that cannot be encoded, either in their natural forms, or as close structural or electronic mimics. Site-specific incorporation of such UAAs would greatly enhance our ability to study natural processes. The inability to create glycoproteins, for example, remains a glaring frustration.

For those wishing to incorporate a novel UAA, it is worth noting that while many aaRS/tRNA pairs have been developed for a specific amino acid of choice and have no observed cross-reactivity with natural amino acids, these may still have a broad substrate specificity, allowing for the incorporation of a number of related UAAs by a single aaRS.^55a,179^ As such, there may be no need to go through the complex process of developing a novel specific system for incorporation of a desired UAA in every case. Rather, it may be possible to utilise an aaRS designed for a structurally related UAA, at least as a starting point from which further manipulations can lead to increased selectivities.^67,179b^

Importantly, though, logistical barriers also exist. In order to facilitate further progress in the field of codon reassignment, it is not only important to explore new techniques, organisms, and orthogonal pairs. It is also vital that the requisite plasmids and constructs become more widely available, as at present gaining access to some variants can be limited and is often a slow and tedious process. Deposition, which is now common for many plasmids, seems to have been slowly embraced by the suppression community.

Finally, perhaps more ambition is needed? Only when more researchers begin to apply their ideas not just to the incorporation of novel UAAs, but to their applications and functions also, can the true potential of this powerful tool be realized. In the future, codon reassignment may have at least a similar impact on the biological sciences as site-directed mutagenesis did before it and given the potential for further selective chemical elaboration in an almost unlimited manner, perhaps even more so.
