# Supplementary material for: Designing logical codon reassignment – Expanding the chemistry in biology
Source: Chem Sci. 2014 Jul 14;6(1):50–69. doi: 10.1039/c4sc01534g (PMC5424465; doi:10.1039/c4sc01534g)
Supplement: Supplementary file 1 [file SC-006-C4SC01534G-s001.pdf]

Electronic Supplementary Material (ESI) for Chemical Science.
This journal is © The Royal Society of Chemistry 2014

Designing logical codon reassignment – expanding the chemistry in biology

Anaëlle Dumas,ϕ[a] Lukas Lercher,ϕ[a] Chris Spicer,ϕ[a] and Benjamin G. Davis*[a]

SUPPLEMENTARY INFORMATION

tRNA-RS pair	Specification (mutations)	a.a. Name	a.a. Structure	Organism	Applications	Misc.	Group	Year	Journal	doi
**Prokaryotic or eukaryotic cell culture**										
**MjTyrRS**										
MjTyrRS / MjtRNATyr	Y32Q D158A E107T L162P	*p*-Methoxy-*L*-phenylalanine	**1** 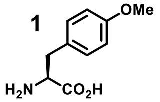	*E. coli*	First UAA incorporation by amber suppression		Schultz	2001	Science	10.1126/science.1060077
MjTyrRS / MjtRNATyr	E107A D158C I159A	*O*-Allyl-*L*-tyrosine	**2** 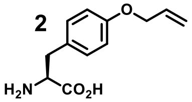	*E. coli*	Speculated cross-coupling		Schultz	2002	Angew. Chem. Int. Edit.	10.1002/1521-3773(20020802)41:15<2840::AID-ANIE2840>3.0.CO;2-#
MjTyrRS / MjtRNATyr	Y32S E107T D158T I159Y L162A	*O*-Allyl-*L*-tyrosine	**2** 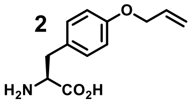	*E. coli*		Paper develops fluorescent method for rapid isolation of AARS/tRNA pairs	Schultz	2002	Nat. Biotechnol.	10.1038/nbt742
MjTyrRS / MjtRNATyr	Y32L D158P I159A L162Q A167V	3-(2-Naphthyl)-*L*-alanine	**3** 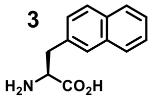	*E. coli*	Speculated novel stacking properties		Schultz	2002	J. Am. Chem. Soc.	10.1021/ja012307j
MjTyrRS / MjtRNATyr	H70A D158T	3-Iodo-*L*-tyrosine	**4** 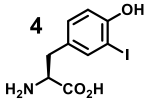	*E. coli*	Aid protein crystal structure deciphering		Yokoyama	2009	Structure	10.1016/j.str.2009.01.008
MjTyrRS / MjtRNATyr	Y32G E107S D158T I159S	*p*-Benzoyl-*L*-phenylalanine (*p*Bpa)	**5** 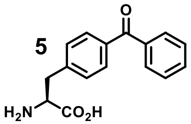	*E. coli*	Photo-cross-linker	Used to cross-link GST mutants at PPI	Schultz	2002	Proc. Natl. Acad. Sci.	10.1073/pnas.172226299
MjTyrRS / MjtRNATyr	A31V Y32G E107P D158S I159S	*p*-Benzoyl-*L*-phenylalanine (*p*Bpa)	**5** 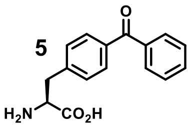	*E. coli*		Demonstration that one synthetase can have a rationally designed, broad scope	Mehl	2009	Mol. Biosyst.	10.1039/b904032c

MjTyrRS / MjtRNATyr	Y32G E107S D158T I159S	*p*-Benzoyl-*L*-phenylalanine (*p*Bpa)	5 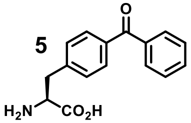	*M. smegmatis*	Amber suppression in a TB model mycobacterium		Schultz	2010	Plos One	10.1371/journal.pone.0009354
MjTyrRS / MjtRNATyr	Y32T E107N D158P L162Q	*p*-Azido-*L*-phenylalanine	6 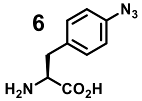	*M. smegmatis*	Amber suppression in a TB model mycobacterium		Schultz	2010	Plos One	10.1371/journal.pone.0009354
MjTyrRS / MjtRNATyr	Y32T E107N D158P L162Q	*p*-Azido-*L*-phenylalanine	6 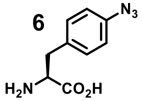	*E. coli*	Photo-cross-linker	Poorer yields for cross-linking than pBpa	Schultz	2002	J. Am. Chem. Soc.	10.1021/ja027007w
MjTyrRS / MjtRNATyr	Y32T E107T D158P I159L L162A	*p*-Amino-*L*-phenylalanine (*p*AF)	7 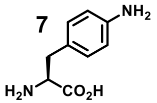	*E. coli*		Paper develops fluorescent method for rapid isolation of AARS/tRNA pairs	Schultz	2002	Nat. Biotechnol.	10.1038/nbt742
MjTyrRS / MjtRNATyr	Y32T E107T D158P I159L L162A	*p*-Amino-*L*-phenylalanine (*p*AF)	7 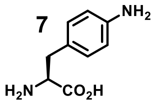	*E. coli*		Bacteria engineered to generate *p*AF biosynthetically. Later used as reactive handle.	Schultz	2003	J. Am. Chem. Soc.	10.1021/ja0284153
MjTyrRS / MjtRNATyr	Y32G T102C V103A E107P D158G I159Y	*p*-Isopropyl-*L*-phenylalanine (*p*IF)	8 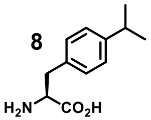	*E. coli*		Paper develops fluorescent method for rapid isolation of AARS/tRNA pairs	Schultz	2002	Nat. Biotechnol.	10.1038/nbt742
MjTyrRS / MjtRNATyr	Y32L D158G I159C L162R	*p*-Acetyl-*L*-phenylalanine	10 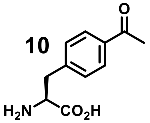	*E. coli*	Labelling with hydrazides *in vitro*		Schultz	2003	Proc. Natl. Acad. Sci.	10.1073/pnas.0234824100
MjTyrRS / MjtRNATyr	Y32A E107P L110F D158A L162A	*O*-Propargyl-*L*-tyrosine	11 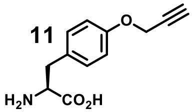	*E. coli*	Reactive handle for azide-alkyne cycloaddition		Schultz	2005	Bioorg. Med. Chem. Lett.	10.1016/j.bmcl.2004.12.065
MjTyrRS / MjtRNATyr	Y32L E107S D158P I159L H160N L162E	*p*-Nitro-*L*-phenylalanine (*p*NO_2Phe)	12 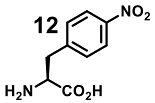	*E. coli*	Fluorescence quencher of Trp, used as a distance probe		Schultz	2006	J. Am. Chem. Soc.	10.1021/ja058262u

MjTyrRS / MjtRNATyr	Y32L<br>E107S<br>D158P<br>I159L<br>H160N<br>L162E	*p*-Nitro-*L*-phenylalanine (pNO_2Phe)	12 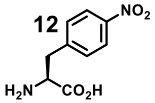	*M. smegmatis*	Amber suppression in a TB model mycobacterium		Schultz	2010	Plos One	10.1371/journal.pone.0009354
MjTyrRS / MjtRNATyr	Y32L<br>L65P<br>D158G<br>I159C<br>L162K	Sulfo-*L*-tyrosine	13 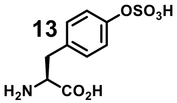	*E. coli*	Natural PTM		Schultz	2006	Nat. Biotechnol.	10.1038/nbt1254
MjTyrRS / MjtRNATyr	Y32G<br>L65E<br>F108A<br>Q109E<br>D158G<br>L162H	Phe-4'-azobenzene (AzoPhe)	14 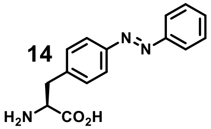	*E. coli*	Photo-isomerizable, used to block binding pockets		Schultz	2006	J. Am. Chem. Soc.	10.1021/ja055467u
MjTyrRS / MjtRNATyr	Y132G<br>F65V<br>F108T<br>D158G<br>L162S		16 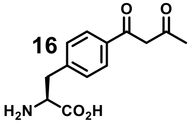	*E. coli*	Labelling with hydroxylamines		Schultz	2006	Bioorg. Med. Chem. Lett.	10.1016/j.bmcl.2006.07.094
MjTyrRS / MjtRNATyr	Y32L<br>L65V<br>F108W<br>Q109M<br>D158G	*p*-Cyano-*L*-phenylalanine (*p*CNPhe)	17 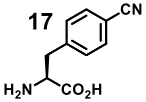	*E. coli*	Infrared probe		Schultz	2006	J. Am. Chem. Soc.	10.1021/ja0636690
MjTyrRS / MjtRNATyr	Y32L<br>L65V<br>F108W<br>Q109M<br>D158G<br>I159P	*p*-Cyano-*L*-phenylalanine	17 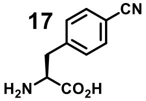	*E. coli*	FRET Probe		Brewer/Mehl	2009	Biochemistry	10.1021/bi900426d
MjTyrRS / MjtRNATyr	Y32Q<br>L65E<br>F108G<br>Q109L<br>L162Y	3-amino-*L*-tyrosine (NH_2Y)	18 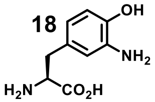	*E. coli*	Used to probe radical propagation in ribonucleotide reductase		Stubbe	2007	J. Am. Chem. Soc.	10.1021/ja076043y
MjTyrRS / MjtRNATyr	Y32S<br>L65A<br>F108K<br>Q109H<br>D158G<br>L162K	*p*-Carboxymethyl-*L*-phenylalanine (pCMF)	19 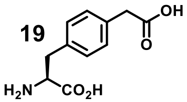	*E. coli*	Stable phosphotyrosine mimic	Racemic a.a. used	Schultz	2007	ACS Chem. Biol.	10.1021/cb700083w

MjTyrRS / MjtRNATyr	Y32I<br>H70F<br>E107S<br>Q109M<br>D158P<br>I159L<br>L162E	TfmdPhe	20 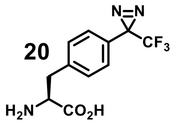	*E. coli*	Photo-cross-linker		Schultz	2007	ChemBioChem	10.1002/cbic.200700460
MjTyrRS / MjtRNATyr	Y32H<br>L65H<br>H70H<br>F108W<br>Q109M<br>Q155Q<br>D158G<br>L162K	BiphenylAla (BipAla)	21 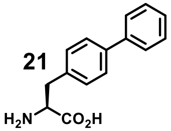	*E. coli*		Synthetase intermediate developed on way to BpyAla incorporation	Schultz	2007	Angew. Chem. Int. Edit.	10.1002/anie.200703397
MjTyrRS / MjtRNATyr	Y32G<br>L65Y<br>H70A<br>F108F<br>Q109Q<br>Q155E<br>D158G<br>I159W<br>L162S	(2,2'-Bipyridin-5-yl)Ala (BpyAla)	22 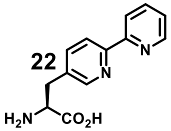	*E. coli*	Metal chelator (specifically Cu^{2+})		Schultz	2007	Angew. Chem. Int. Edit.	10.1002/anie.200703397
MjTyrRS / MjtRNATyr	A31V<br>Y32G<br>E107P<br>D158S<br>I159S	4-Fluoro-*p*-benzoyl-*L*-phenylalanine (4F-Bpa)	23 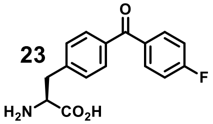	*E. coli*		Demonstration that one synthetase can have a rationally designed broad scope	Mehl	2009	Mol. Biosyst.	10.1039/b904032c
MjTyrRS / MjtRNATyr	Y32V<br>L65M<br>H70T<br>F108R<br>Q109E<br>D158S<br>I159S	HQ-Ala	24 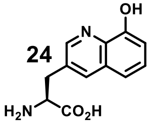	*E. coli*	Metal chelator (specifically zinc $^{2+}$). Also fluorescent		Wang/Schultz	2009	J. Am. Chem. Soc.	10.1021/ja808340b
MjTyrRS / MjtRNATyr	Y32G<br>E107P<br>D158T<br>I159S	2,6-Difluoro-*p*-benzoylPhe (2,6dF-Bpa)	25 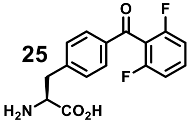	*E. coli*		Demonstration that one synthetase can have a rationally designed broad scope	Mehl	2009	Mol. Biosyst.	10.1039/b904032c

MjTyrRS / MjtRNATyr	Y32S<br>L65A<br>H70M<br>D158S<br>I159E	*p*-Borono-*L*-phenylalanine	26 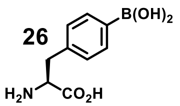	*E. coli*	Used for protein purification, diol conjugation, Suzuki coupling and as latent tyrosine residue		Schultz	2008	Angew. Chem. Int. Edit.	10.1002/anie.200803240
MjTyrRS / MjtRNATyr	Y32G<br>E107P<br>D158T<br>I159S<br>V164A	4-Iodo-*p*-benzoyl-*L*-phenylalanine (4I-Bpa)	27 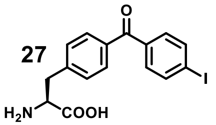	*E. coli*		Demonstration that one synthetase can have a rationally designed broad scope	Mehl	2009	Mol. Biosyst.	10.1039/b904032c
MjTyrRS / MjtRNATyr	Y32G<br>E107P<br>D158T<br>I159S<br>V164A	4-Nitro-*p*-benzoyl-*L*-phenylalanine (4Nitro-Bpa)	28 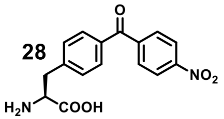	*E. coli*		Demonstration that one synthetase can have a rationally designed broad scope	Mehl	2009	Mol. Biosyst.	10.1039/b904032c
MjTyrRS / MjtRNATyr	Y32G<br>E107P<br>D158T<br>I159S<br>V164A	3-Fluoro-4-nitro-*p*-benzoyl-*L*-phenylalanine (3F-4nitro-Bpa)	29 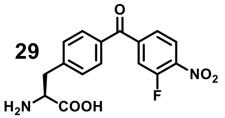	*E. coli*		Demonstration that one synthetase can have a rationally designed broad scope	Mehl	2009	Mol. Biosyst.	10.1039/b904032c
MjTyrRS / MjtRNATyr	A31V<br>Y32G<br>E107P<br>D158S<br>I159S	Thyronine	30 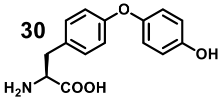	*E. coli*		Demonstration that one synthetase can have a rationally designed broad scope	Mehl	2009	Mol. Biosyst.	10.1039/b904032c
MjTyrRS / MjtRNATyr	Y32G<br>E107P<br>D158T<br>I159S	*O*-Benzyl-*L*-tyrosine	31 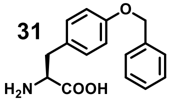	*E. coli*		Demonstration that one synthetase can have a rationally designed broad scope	Mehl	2009	Mol. Biosyst.	10.1039/b904032c
MjTyrRS / MjtRNATyr	Y32G<br>L65G<br>H70N<br>F108G<br>D158S<br>I159M<br>L162N	*o*-Nitrobenzyl-2-fluoro-*L*-tyrosine	32 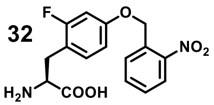	*E. coli*	Photo-caged fluoro-tyrosine		Cropp/ Deiters	2010	Biochemistry	10.1021/bi100013s

MjTyrRS / MjtRNATyr	Y32G L65H A67G H70G F108L Q109S Y114S D158T I159Y L162D	2-Nitro-*L*-phenylalanine	**33** 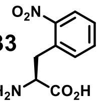	*E. coli*	Photo-cleavage of protein backbone	Attempted to incorporate NitroGly but unsuccesful	Schultz	2009	Chem. Biol.	10.1016/j.chembiol.2009.01.013
MjTyrRS / MjtRNATyr	Y32A L65S F108Q H109A D158A L162Y	*p*-OCF_3-Phe	**34** 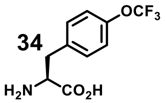	*E. coli*	^{19}F Probe for protein NMR	Development of an inducible system for aaRS expression	Geierstanger/ Schultz	2008	J. Am. Chem. Soc.	10.1021/ja801602q
MjTyrRS / MjtRNATyr	Q155R Q173G I176V	*p*-Hydroxy-phenyllactic acid	**35** 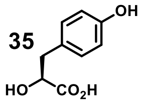	*E. coli*	Hydroxy acid to introduce ester linkages into protein backbone		Schultz	2008	Angew. Chem. Int. Edit.	10.1002/ange.200704074
MjTyrRS / MjtRNATyr	Y32R L65L H70L Q155M D158G I159L L162H	3-Nitro-*L*-tyrosine	**36** 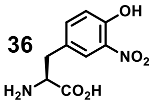	*E. coli*	Genetically encoded marker of protein oxidative damage		Chin/ Mehl	2008	J. Am. Chem. Soc.	10.1021/ja710100d
MjTyrRS / MjtRNATyr	Y32T E107T D158P I159L L162A	*p*AMF	**37** 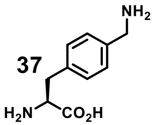	*E. coli*	Improving enzyme activity of a nitroreductase		Mehl	2006	J. Am. Chem. Soc.	10.1021/ja061099y
MjTyrRS / MjtRNATyr	Y32L L65A F108S H109H D158A L162M	*p*-Methyl-*L*-phenylalanine	**38** 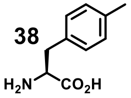	*E. coli*	Improving enzyme activity of a nitroreductase		Mehl	2006	J. Am. Chem. Soc.	10.1021/ja061099y
MjTyrRS / MjtRNATyr	Y32L A67S H70N A167Q	3,4-Dihydroxy-*L*-phenylalanine	**39** 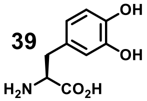	*E. coli*	Redox active amino acid		Schultz	2003	J. Am. Chem. Soc.	10.1021/ja038242x

MjTyrRS / MjtRNATyr	Y32L<br>L65V<br>F108W<br>Q109M<br>D158G<br>I159P	*p*-Ethynyl-*L*-phenylalanine	41 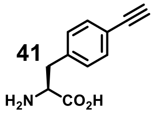	*E. coli*	FRET Probe		Brewer/Mehl	2009	Biochemistry	10.1021/bi900426d
MjTyrRS / MjtRNATyr	Y32L<br>L65A<br>F108S<br>H109H<br>D158A<br>L162M	Trifluoromethyl-*L*-phenylalanine (tfmPhe)	42 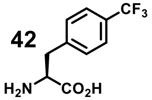	*E. coli*	^{19}F Probe for protein NMR		Mehl	2007	J. Am. Chem. Soc.	10.1021/ja064661t
MjTyrRS / MjtRNATyr	Y32G<br>L65G<br>F108E<br>D158S<br>L162E	*o*-Nitrobenzyl-*O*-tyrosine (ONBY)	43 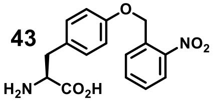	*E. coli*	Photocaged tyrosine		Schultz	2006	Angew. Chem. Int. Edit.	10.1002/anie.200600264
MjTyrRS / MjtRNATyr		*p*-Bromo-*L*-phenylalanine	44 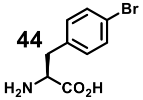	*E. coli*	Mutant GFP excitation/ emission		Schultz	2003	J. Org. Chem.	10.1021/jo02657u
MjTyrRS / MjtRNATyr	Y32L<br>E107S<br>D158P<br>I159L<br>L162E	*p*-Iodo-*L*-phenylalanine	45 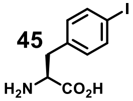	*E. coli*	Mutant GFP excitation/ emission, later used for protein crystallisation and as reactive handle		Schultz	2003	J. Org. Chem.	10.1021/jo02657u
MjTyrRS / MjtRNATyr	Y32L<br>E107S<br>D158P<br>I159L<br>L162E	*p*-Iodo-*L*-phenylalanine	45 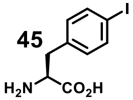	*E. coli*	Structural determination of proteins		Schultz	2003	Nat. Biotechnol.	10.1038/nbt1013
MjTyrRS / MjtRNATyr	Y32L<br>E107S<br>D158P<br>I159L<br>L162E	*p*-Iodo-*L*-phenylalanine	45 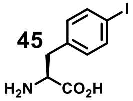	*M. smegmatis*	Amber suppression in a TB model mycobacterium		Schultz	2010	Plos One	10.1371/journal.pone.0009354
MjTyrRS / MjtRNATyr	Y32G<br>L65G<br>H70N<br>F108G<br>D158S<br>I159M<br>L162N	*o*-Nitrobenzyl-3-fluoro-*L*-tyrosine	46 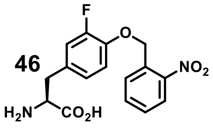	*E. coli*	Photocaged fluoroTyr		Cropp/ Deiters	2010	Biochemistry	10.1021/bi100013s

MjTyrRS / MjtRNATyr	Y32G<br>L65G<br>H70M<br>F108G<br>D158S<br>I159M<br>L162E	*o*-Nitrobenzyl-2,6-difluoro-*L*-tyrosine	47 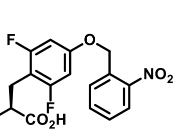	*E. coli*	Photocaged fluoroTyr		Cropp/ Deiters	2010	Biochemistry	10.1021/bi100013s
MjTyrRS / MjtRNATyr	Y32L<br>A67S<br>H70N<br>A167Q	PhenylselenoCys	48 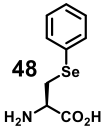	*E. coli*	Precursor of Dha		Schultz	2007	Angew. Chem. Int. Edit.	10.1002/ange.200702305
MjTyrRS / MjtRNATyr	Y32L<br>D158E<br>I159P<br>H160Q<br>Y161G<br>L162R<br>G163D	*m*-Acetyl-*L*-phenylalanine	51 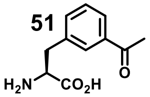	*E. coli*	Labelling with hydrazides, both *in vitro* and on cell surfaces	Coupling efficiencies comparable to *p*-derivative. First labelling on cell surface by amber suppression	Schultz	2003	Biochemistry	10.1021/bi0300231
MjTyrRS / MjtRNATyr	Y32E<br>L65H<br>A67G<br>H70G<br>F108Y<br>Q109H<br>D158G<br>L162G	7-MethylCoumarinyl-ethylGly	52 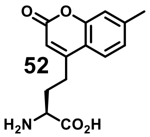	*E. coli*	Improving enzyme activity of a phosphotriesterase		Jackson	2011	J. Am. Chem. Soc.	10.1021/ja106416g
MjTyrRS / MjtRNATyr	Y32E<br>L65H<br>A67G<br>H70G<br>F108Y<br>Q109H<br>D158G<br>L162G	(7-Hydroxycoumarin-4-yl)ethylGly	53 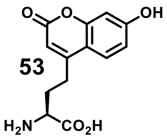	*E. coli*	Fluorescent amino acid used to probe unfolding, and later used to improve enzyme activity		Schultz	2006	J. Am. Chem. Soc.	10.1021/ja062666k
MjTyrRS / MjtRNATyr	Y32E<br>L65A<br>A107E<br>F108P<br>Q109S<br>D158G<br>L162G	4-(6-methyl-s-tetrazin-3-yl)amino-*L*-phenylalanine<br>.	54 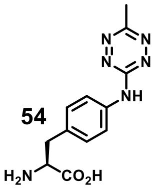	*E. coli*	Reactive handle for tetrazine ligation		Mehl	2012	J. Am. Chem. Soc.	10.1021/ja2109745

MjTyrRS / MjtRNATyr	Y32I<br>L65I<br>Q109M<br>D158G<br>L162V<br>V164G	*p*-(2-tetrazole) -*L*-phenylalanine	55 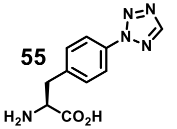	*E. coli*	Reactive handle for 'Photo-click' reaction		Lin	2010	J. Am. Chem. Soc.	10.1021/ja104350y
MjTyrRS / MjtRNATyr	Y32V<br>L65Y<br>F108H<br>Q109G<br>D158G<br>L162E<br>D286R	*p*-Acrylamido-(*S*)-*L*-phenylalanine (AcrF)	56 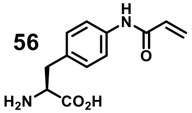	*E. coli*	Reactive handle for Michael additions, cross-linker		Schultz / Kim	2014	J. Am. Chem. Soc.	10.1021/ja502851h
MjTyrRS / MjtRNATyr	Y32G<br>L65Y<br>F108H<br>Q109G<br>D158G<br>I159L<br>L162Q<br>D286R	*p*-Vinylsulfonamido-(*S*)-*L*-phenylalanine (VSF)	57 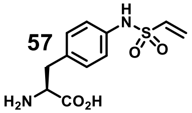	*E. coli*	Reactive handle for Michael additions, cross-linker		Schultz / Kim	2014	J. Am. Chem. Soc.	10.1021/ja502851h
MjTyrRS / MjtRNATyr	Y32E<br>L65T<br>D158S<br>I159A<br>H160P<br>Y161T<br>L162Q<br>A167W<br>D286R<br>or<br>Y32E<br>L65V<br>K90E<br>I159A<br>H160W<br>Y161G<br>L162Q<br>A167I<br>D286R	2-amino-3-(6-hydroxy-2-naphthyl)-propanoic acid (NpOH)	134 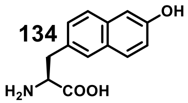	*E. coli*	Reactive handle for azo coupling		Tsao	2013	Bioconjugate Chem.	10.1021/bc400168u

EcTyrRS										
E. coli TyrRS/tRNA	Y37V<br>D182S<br>F183M	*p*-Methoxy-*L*-phenylalanine	1 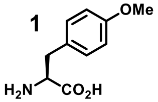	*S. cerevisiae*		Range of UAA's incorporated into yeast cells	Schultz	2003	Science	10.1126/science.1084772
E. coli TyrRS/*B. Steareothermophilus* tRNATyr	Y37V<br>Q195C	3-Iodo-*L*-tyrosine	4 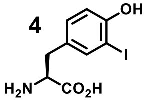	Mammalian cells (CHO)	Speculated reactive handle, thyroid modelling	First UAA Incorporation in mammalian cells by amber suppression	Yokoyama	2002	Nucleic Acid Res.	10.1093/nar/gkf589
E. coli TyrRS/tRNA	H70A<br>D158T	3-Iodo-*L*-tyrosine	4 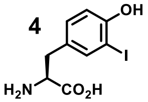	*E. coli*		*E. coli* wt Tyr synthetase replaced by *M. jann.*, and then mutant *E. coli* TyrRS used to incorporate UAA's	Sakamoto	2010	Nucleic Acid Res.	10.1093/nar/gkq080
E. coli TyrRS/tRNA	Y37G<br>D182G<br>L186A	*p*-Benzoyl-*L*-phenylalanine (*p*Bpa)	5 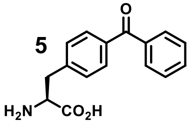	Neural Stem Cells		First UAA incorporation in neural stem cells	Wang	2011	Stem Cells	10.1002/stem.679
E. coli TyrRS/tRNA	Y37G<br>D182G<br>L186A	*p*-Benzoyl-*L*-phenylalanine (*p*Bpa))	5 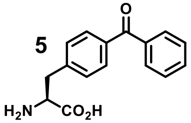	*S. cerevisiae*		Range of UAA's incorporated into yeast cells	Schultz	2003	Science	10.1126/science.1084772
E. coli TyrRS/tRNA	Y37L<br>D182S<br>F183M<br>L186A	*p*-Azido *L*-phenylalanine	6 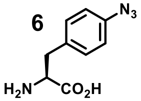	*S. cerevisiae*		Range of UAA's incorporated into yeast cells	Schultz	2003	Science	10.1126/science.1084772
E. coli TyrRS/tRNA	Y37I<br>D182G<br>F183M<br>L186A	*p*-Acetyl-*L*-phenylalanine	10 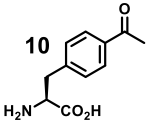	*S. cerevisiae*		Range of UAA's incorporated into yeast cells	Schultz	2003	Science	10.1126/science.1084772
E. coli TyrRS/tRNA	Y37V<br>D182S<br>F183Y	*p*-Iodo-*L*-phenylalanine	45 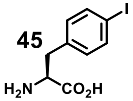	*S. cerevisiae*		Range of UAA's incorporated into yeast cells	Schultz	2003	Science	10.1126/science.1084772
E. coli TyrRS/tRNA	H70A<br>D158T	3-Azido-*L*-tyrosine	75 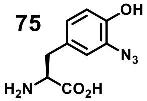	*E. coli*		*E. coli* wt Tyr synthetase replaced by *M. Jann.*, and then mutant *E. coli*	Sakamoto	2010	Nucleic Acid Res.	10.1093/nar/gkq080

						TyrRS used to incorporate UAA's				
***E. coli* LeuRS**										
E. coli LeuRS/tRNA	M40L<br>L41E<br>Y499R<br>Y527A<br>H537G	*O*-Methyl-*L*-tyrosine	1 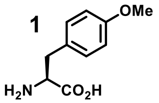	*S. cerevisiae*			Schultz	2004	J. Am. Chem. Chem. Soc.	10.1021/ja040175z
E. coli LeuRS/tRNA	M40W<br>L41S<br>Y499I<br>Y527A<br>H537G	*O*-Nitrobenzyl-*L*-cysteine	15 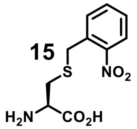	*S. cerevisiae*	Photocaged cysteine		Schultz	2004	J. Am. Chem. Chem. Soc.	10.1021/ja040175z
E. coli LeuRS/tRNA	M40A<br>L41N<br>T252A<br>Y499I<br>Y527G<br>H537T	DansylAla	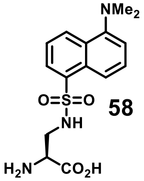 58	*S. cerevisiae*	Fluorescent amino acid		Schultz	2006	Proc. Natl. Acad. Sci.	10.1073/pnas.0603965103
E. coli LeuRS/tRNA	M40A<br>L41N<br>Y499I<br>Y527G<br>H537T	DansylAla	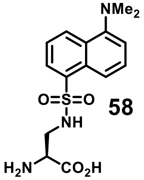 58	Neural Stem Cells	Fluorescence measuring of ion channels	First UAA incorporation in neural stem cells	Wang	2011	Stem Cells	10.1002/stem.679
E. coli LeuRS/tRNA	M40G<br>L41Q<br>T252A<br>Y499L<br>Y527G<br>H537F	4,5-Dimethoxy-2-nitrobenzylserine	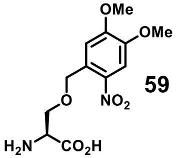 59	*S. cerevisiae*	Photocaged serine		Schultz	2007	Nat. Chem. Biol	10.1038/nchembio.2007.44
E. coli LeuRS/tRNA	E20K<br>M40V<br>L41S<br>T252R<br>Y499S<br>Y527L<br>H537G		60 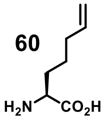	*S. cerevisiae*	Reactive handle e.g. for olefin metathesis		Schultz	2010	Angew. Chem. Int. Edit.	10.1002/anie.200905590
E. coli LeuRS/tRNA	M40V<br>L41M<br>Y499L	α-Aminocaprylic acid	61 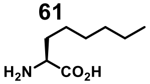	*S. cerevisiae*			Schultz	2004	J. Am. Chem. Chem. Soc.	10.1021/ja040175z

	Y527L<br>H537G									
E. coli LeuRS / EctRNALeu	M40I<br>Y499I<br>Y527A<br>H537G	2-Aminocaprylic acid	61 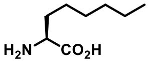	*S. cerevisiae*		Probable background incorporation of Leu, Gln, Thr, Lys	Schultz	2008	Bioorg. Med. Chem. Lett.	10.1016/j.bmcl.2008.09.050
E. coli LeuRS / EctRNALeu	M40<br>Y499I<br>Y527A<br>H537G	2-Aminononanoic acid	62 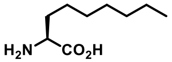	*S. cerevisiae*		Probable background incorporation of Leu, Gln, Thr, Lys	Schultz	2008	Bioorg. Med. Chem. Lett.	10.1016/j.bmcl.2008.09.050
E. coli LeuRS / EctRNALeu	M40I<br>Y499I<br>Y527A<br>H537G	2-Aminodecanoic acid	63 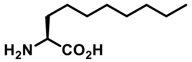	*S. cerevisiae*		Probable background incorporation of Leu, Gln, Thr, Lys	Schultz	2008	Bioorg. Med. Chem. Lett.	10.1016/j.bmcl.2008.09.050
E. coli LeuRS / EctRNALeu	M40I<br>Y499I<br>Y527A<br>H537G		64 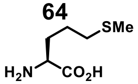	*S. cerevisiae*		Probable background incorporation of Leu, Gln, Thr, Lys	Schultz	2008	Bioorg. Med. Chem. Lett.	10.1016/j.bmcl.2008.09.050
E. coli LeuRS / EctRNALeu	M40I<br>Y499I<br>Y527A<br>H537G		65 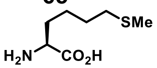	*S. cerevisiae*		Probable background incorporation of Leu, Gln, Thr, Lys	Schultz	2008	Bioorg. Med. Chem. Lett.	10.1016/j.bmcl.2008.09.050
E. coli LeuRS / EctRNALeu	M40I<br>Y499I<br>Y527A<br>H537G		66 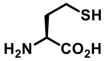	*S. cerevisiae*		Probable background incorporation of Leu, Gln, Thr, Lys	Schultz	2008	Bioorg. Med. Chem. Lett.	10.1016/j.bmcl.2008.09.050
E. coli LeuRS / EctRNALeu	M40I<br>Y499I<br>Y527A<br>H537G		67 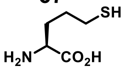	*S. cerevisiae*		Background incorporation of Leu, Gln, Thr, Lys	Schultz	2008	Bioorg. Med. Chem. Lett.	10.1016/j.bmcl.2008.09.050
E. coli LeuRS / EctRNALeu	M40I<br>Y499I<br>Y527A<br>H537G		68 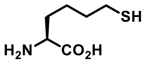	*S. cerevisiae*		Background incorporation of Leu, Gln, Thr, Lys	Schultz	2008	Bioorg. Med. Chem. Lett.	10.1016/j.bmcl.2008.09.050
E. coli LeuRS/tRNA	E20K<br>M40V<br>L41S<br>T252R<br>Y499S<br>Y527L<br>H537G	Alliin	69 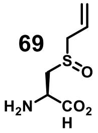	*S. cerevisiae*	Reactive handle e.g. for olefin metathesis		Schultz	2010	Angew. Chem. Int. Edit.	10.1002/anie.200905590

E. coli LeuRS/tRNA	E20K<br>M40V<br>L41S<br>T252R<br>Y499S<br>Y527L<br>H537G		70 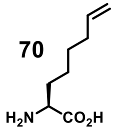	*S. cerevisiae*	Reactive handle e.g. for olefin metathesis		Schultz	2010	Angew. Chem. Int. Edit.	10.1002/anie.200905590
E. coli LeuRS/tRNA	E20K<br>M40V<br>L41S<br>T252R<br>Y499S<br>Y527L<br>H537G		71 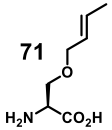	*S. cerevisiae*	Reactive handle e.g. for olefin metathesis		Schultz	2010	Angew. Chem. Int. Edit.	10.1002/anie.200905590
E. coli LeuRS/tRNA	E20K<br>M40V<br>L41S<br>T252R<br>Y499S<br>Y527L<br>H537G		72 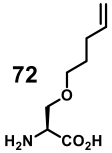	*S. cerevisiae*	Reactive handle e.g. for olefin metathesis		Schultz	2010	Angew. Chem. Int. Edit.	10.1002/anie.200905590
E. coli LeuRS/tRNA	L38F<br>M40G<br>L41P<br>Y499V<br>Y500L<br>Y527A<br>H537G5<br>L538S<br>F541C<br>A560V	3-(6-Acetylnaphthalen-2-ylamino)-2-aminopropanoic acid (Anap)	73 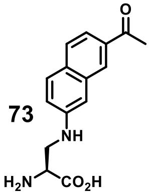	*S. cerevisiae*	Highly environmentally sensitive fluorescent probe		Schultz	2009	J. Am. Chem. Soc.	10.1021/ja904896s
E. coli LeuRS/tRNA	M40G<br>L41P<br>Y499G<br>Y527A<br>H537T	3-(Naphthalen-2-ylamino)-2-aminopropanoic acid (Nap)	74 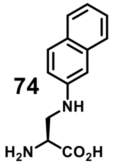	*S. cerevisiae*		Synthetase intermediate developed on way to Anap incorporation	Schultz	2009	J. Am. Chem. Soc.	10.1021/ja904896s

**Mm/Mb PylRS**										
MmPylRS / MmtRNAPyl	N346A C348A	*p*-Methoxy-*L*-phenylalanine	1 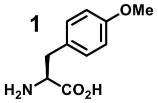	*E. coli*		Low incorporation efficiency. This mutant does not incorporate phenylalanine analogues with small ortho-substituents	Liu	2012	J. Am. Chem. Soc.	10.1021/ja211972x
MmPylRS / MmtRNAPyl	A302T N346T C348T	*p*-Benzoyl-*L*-phenylalanine (*p*Bpa)	5 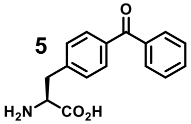	*E. coli*		Improved evolution strategy (small-intelligent mutagenesis)	Wang	2013	ChemBioChem	10.1002/cbic.201300400
MmPylRS / MmtRNAPyl	N346A C348L	Phenylalanine	9 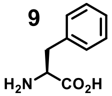	*E. coli*			Liu	2011	Mol. Biosyst.	10.1039/c0mb00217h
MmPylRS / MmtRNAPyl	N346A C348A	*O*-propargyl-*L*-tyrosine	11 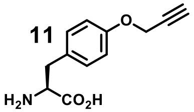	*E. coli*	Reactive handle for azide-alkyne cycloaddition		Liu	2012	J. Am. Chem. Soc.	10.1021/ja211972x
MmPylRS / MmtRNAPyl	N346A C348A	*O*-Benzyl-*L*-tyrosine	31 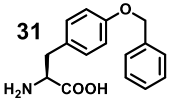	*E. coli*			Liu	2012	J. Am. Chem. Soc.	10.1021/ja211972x
MmPylRS / MmtRNAPyl	L305M Y306L L309S N346S C348M	*p*-Bromo-*L*-phenylalanine	44 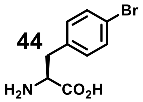	*E. coli*			Liu	2011	Mol. Biosyst.	10.1039/c0mb00217h
MmPylRS / MmtRNAPyl	L305M Y306L L309S N346S C348M	*p*-Iodo-*L*-phenylalanine	45 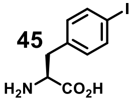	*E. coli*			Liu	2011	Mol. Biosyst.	10.1039/c0mb00217h
MmPylRS / MmtRNAPyl	N346A C348A	*O*-*tert*-butyl-*L*-tyrosine	50 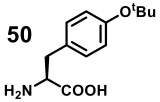	*E. coli*			Liu	2012	J. Am. Chem. Soc.	10.1021/ja211972x
MmPylRS / MmtRNAPyl	N346A C348A	*m*-Acetyl-*L*-phenylalanine	51 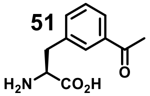	*E. coli*	Reactive handle for oxime formation		Liu	2013	ACS Chem. Biol.	10.1021/cb300512r

MmPylRS / MmtRNAPyl	R61K G131E L309A C348V Y384F	*N*[ε]-Benzyloxycarbonyl-lysine (ZLys)	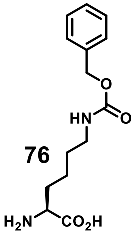 76	Mammalian cells (HEK), *E. coli* and *S. cerevisiae*		Evolved in *E. coli*	Yokoyama	2008	Biochem. Biophys. Res. Comm.	10.1016/j.bbrc.2008.04.164
MmPylRS / MmtRNAPyl	Y306A Y384F	*N*[ε] -Benzyloxycarbonyl-*L*-lysine (ZLys)	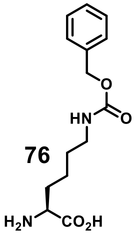 76	*E. coli*			Yokoyama	2008	Chem. Bio.	10.1016/j.chembiol.2008.10.004
MmPylRS / MmtRNAPyl	Y384F	*N*[ε]-(*tert*-Butoxycarbonyl)-*L*-lysine (BocLys)	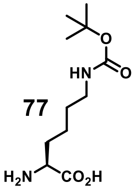 77	*E. coli*			Yokoyama	2008	Chem. Bio.	10.1016/j.chembiol.2008.10.004
MmPylRS / MmtRNAPyl	R61K, G131E, Y384F	*N*[ε]-(*tert*-Butoxycarbonyl)-*L*-lysine (BocLys)	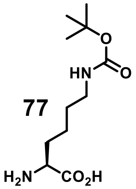 77	*S. cerevisiae*			Yokoyama	2008	Biochem. Biophys. Res. Comm.	10.1016/j.bbrc.2008.04.164
MbPylRS / MbtRNAPyl	Wild type	ε-*N,N*-Dimethyl-*L*-lysine	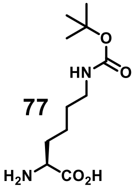 77	*E. coli*	Precursor to *N,N*-dimethyllysine		Chin	2010	Chem. Biol.	10.1016/j.chembiol.2010.07.013

MmPylRS / MmtRNAPyl	L305I<br>Y306F<br>L309A<br>C348F	N^{ϵ}-Acetyllysine (AcLys)	**78** 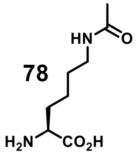	Mammalian cells			Yokoyama	2008	Biochem. Biophys. Res. Comm.	10.1016/j.bbrc. 2008.04.164
MbPylRS / MbtRNAPyl	D76G<br>L266V<br>L270I<br>Y271F<br>L274A<br>C313F	N^{ϵ}-Acetyllysine (AcLys)	**78** 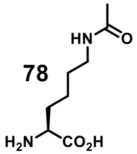	*E. coli*			Chin	2008	Nat. Chem. Biol	10.1038/nchem bio.73
MbPylRS / SctDNA$^{Arg}_{UCU}$- MmtDNA$^{Pyl}_{CUA}$	L266M<br>L270I<br>Y271F<br>L274A<br>C313F	N^{ϵ}-Acetyllysine (AcLys)	**78** 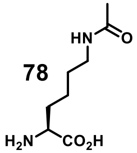	*S. cerevisiae*		Special expression cassette developed for the expression of the tRNA in yeast	Chin	2010	J. Am. Chem. Soc.	10.1021/ja1046 09m
MbPylRS / MbtRNAPyl	Wild type	Pyrrolysine (Pyl)	**79** 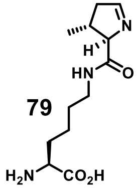	*E. coli*		First incorporation of Pyl in another organism	Krzycki	2004	Nature	10.1038/nature 02895
MmPylRS / MmtRNAPyl	Y306A<br>Y384F	*Nε*-(*o*-Azidobenzyloxycarbonyl)-*L*-lysine (AzZLys)	**80** 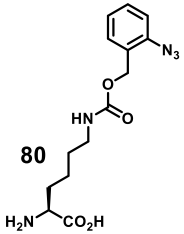	*E. coli*	Reactive handle for Staudinger ligation	Photo-sensitive	Yokoyama	2008	Chem. Bio.	10.1016/j.chem biol.2008.10.00 4
MbPylRS / MbtRNAPyl	Wild type	N^{ϵ} -*D*-Prolyl-*L*-lysine	**81** 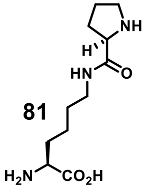	*E. coli*		Does not work with *L*-prolyl analogue	Ambrogelly	2006	FEBS Lett.	10.1016/j.febsle t.2006.11.028

MbPylRS / MbtRNAPyl	Wild type	N^{ε}-Cyclopentyloxycarbonyl-*L*-lysine (Cyc)	82 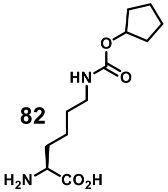	*E. coli*			Ambrogelly	2006	FEBS Lett.	10.1016/j.febslet.2006.11.028
MmPylRS / MmtRNAPyl	Wild type	N^{ε}-Cyclopentyloxycarbonyl-*L*-lysine (Cyc)	82 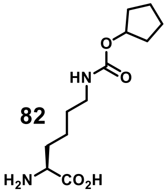	*E. coli*, mammalian cells			Geigerstanger / Schultz	2009	Angew. Chem. Int. Edit.	10.1002/anie.200900683
MbPylRS / MbtRNAPyl	Wild type	2-Amino-6-(cyclopentanecarboxyamino)hexanoic acid (Cpn-Lys)	83 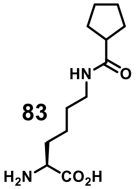	*E. coli*			Chan / Krzycki	2009	J. Mol. Biol.	10.1016/j.jmb.2008.11.032
MbPylRS / MbtRNAPyl	Wild type	2-Amino-6-((*R*)-tetrahydrofuran-2-carboxyamido) hexanoic acid (2Thf-Lys)	84 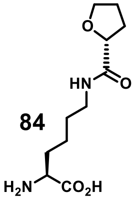	*E. coli*		Does not work with 3Thf-Lys or 4Thf-Lys	Chan / Krzycki	2009	J. Mol. Biol.	10.1016/j.jmb.2008.11.032
MmPylRS / MmtRNAPyl	Wild type		85 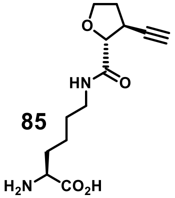	*E. coli*	Reactive handle for azide-alkyne cycloaddition		Chan	2009	Angew. Chem. Int. Edit.	10.1002/anie.200805420

MbPylRS / MbtRNAPyl	Wild type	N^{ε}-[(2-Propynyloxy)carbonyl]-*L*-lysine	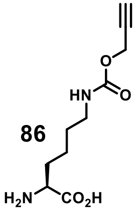 86	*E. coli*	Reactive handle for azide-alkyne cycloaddition	Does not work if carbamate is replaced by amide	Deiters / Chin	2009	J. Am. Chem. Soc.	10.1021/ja900553w
MbPylRS / MbtRNAPyl	L274A C313V Y349F	N^{ε}-[(2-Propynyloxy)carbonyl]-*L*-lysine	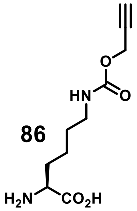 86	*E. Coli*			Guo	2013	Org. Biomol. Chem.	10.1039/c3ob27116a
MbPylRS / $SctDNA^{Arg}_{UCU}$-$MmtDNA^{Pyl}_{CUA}$	Wild type	N^{ε}-[(2-Propynyloxy)carbonyl]-*L*-lysine	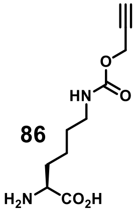 86	*S. cerevisiae*		Special expression cassette developed for the expression of the tRNA in yeast	Chin	2010	J. Am. Chem. Soc.	10.1021/ja104609m
PylRS / tRNAPyl	Wild type	N^{ε}–(((*1R,2R*)-2-azidocyclopentyloxy)carbonyl)-*L*-lysine (ACPK)	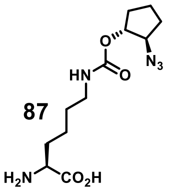 87	Enteropathogenic *E. coli* (EPEC), Shigella, Salmonella	Labelling of bacterial effectors	Unconventional promoter needed in Salmonella	Chen	2011	J. Am. Chem. Soc	10.1021/ja209008w
MbPylRS / MbtRNAPyl	L274A C313V Y349F	N^{ε}–(((*1R,2R*)-2-azidocyclopentyloxy)carbonyl)-*L*-lysine (ACPK)	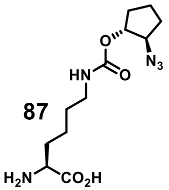 87	*E. coli*, mammalian cells (HEK)	Reactive handle for azide-alkyne cycloaddition	Poor incorporation with wild type. Better incorporation than aliphatic azide	Chen	2011	Chem. Comm	10.1039/c1cc00024a

MbPylRS / MbtRNAPyl	L270F L274M N311G C313G	*o*-Nitrobenzyl-*O*-tyrosine	**88** 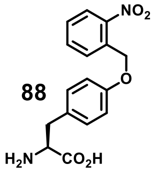	*E. coli*, mammalian cells (HEK)	Photocaged tyrosine to study tyrosine phosphorylation	Evolved in *E. coli*	Chin	2012	J. Am. Chem. Soc	10.1021/ja3046958
MmPylRS / MmtRNAPyl	Y384F Y306A	N^{ε}-Benzyl-oxycarbonyl- *L*-lysine	**89** 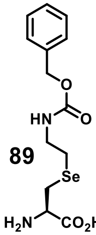	*E. coli*			Liu	2012	Biochemistry	10.1021/bi300535a
MbPylRS / MbtRNAPyl	Wild type		**90** 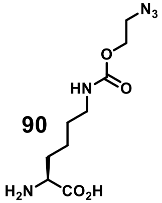	*E. coli*	Reactive handle for azide-alkyne cycloaddition		Deiters / Chin	2009	J. Am. Chem. Soc.	10.1021/ja900553w
MmPylRS / MmtRNAPyl	Y306M L309A C348A Y384F	*o*-Nitrobenzyl-oxycarbonyl-N^{ε}-*L*-lysine	**91** 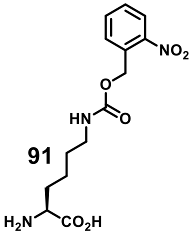	*E. coli,* mammalian cells			Geigerstanger / Schultz	2009	Angew. Chem. Int. Edit.	10.1002/anie.200900683
MmPylRS / MmtRNAPyl	Wild type	*D*-Cys-ε-Lys ("(*S,S*)")	**92** 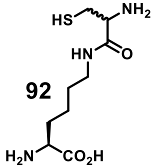	*E. coli*	Reactive handle for NCL	(R,S) incorporation much less efficient	Chan	2009	Angew. Chem. Int. Edit.	10.1002/anie.200904472

MbPylRS / MbtRNAPyl	C313V	N^{ε}-Cysteinyl-*L*-lysine	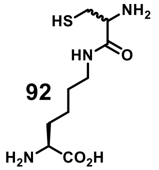 92	*E. coli*	Reactive handle for NCL		Chin	2011	J. Am. Chem. Soc	10.1021/ja203111c
MbPylRS / SctDNA$^{Arg}_{UCU}$-MmtDNA$^{Pyl}_{CUA}$	L270I Y271L L274A C313F	N^{ε}-Trifluoroacetyllysine	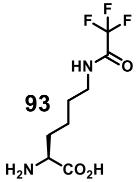 93	*S. cerevisiae*		Special expression cassette developed for the expression of the tRNA in yeast	Chin	2010	J. Am. Chem. Soc.	10.1021/ja104609m
MbPylRS / SctDNA$^{Arg}_{UCU}$-MmtDNA$^{Pyl}_{CUA}$	M241F A267S Y271F L274A C313F	N^{ε}-[(1-(6-Nitrobenzo [d][1,3]dioxol-5yl) ethoxy)carbonyl]-*L*-lysine	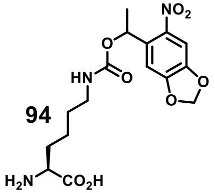 94	*S. cerevisiae*		Special expression cassette developed for the expression of the tRNA in yeast	Chin	2010	J. Am. Chem. Soc.	10.1021/ja104609m
MbPylRS / MbtRNAPyl	M241F A267S Y271C L274M	N^{ε}-[(1-(6-Nitrobenzo [d][1,3]dioxol-5yl) ethoxy)carbonyl]-*L*-lysine	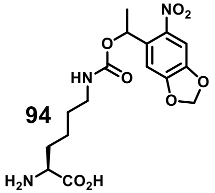 94	Mammalian cells (HEK 293)	Subst. of NLS for photocaged nuclear localization of GFP and p53		Deiters / Chin	2010	J. Am. Chem. Soc.	10.1021/ja910688s
MbPylRS / MbtRNAPyl	M241F A267S Y271C L274M	N^{ε}-[(1-(6-Nitrobenzo [d][1,3]dioxol-5yl) ethoxy)carbonyl]-*L*-lysine	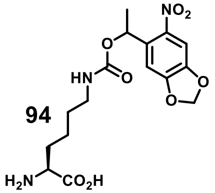 94	Mammalian cells (HEK)	Light-activated gene expression		Deiters	2013	J. Am. Chem. Soc.	10.1021/ja4051026
MbPylRS / SctDNA$^{Arg}_{UCU}$-MmtDNA$^{Pyl}_{CUA}$	Wild type	N^{ε}-[(2-(3-Methyl-3H-diazirin-3-yl)ethoxy)carbonyl]-*L*-lysine	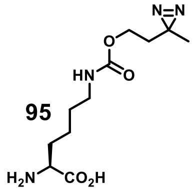 95	Yeast	Photo-cross-linker	Special expression cassette developed for the expression of the tRNA in yeast	Chin	2010	J. Am. Chem. Soc.	10.1021/ja104609m

MbPylRS / MbtRNAPyl	Wild Type	N^{ε}-[(2-(3-Methyl-3H-diazirin-3-yl)ethoxy)carbonyl]-*L*-lysine	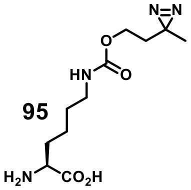 95	*E. coli*, mammalian cells (HEK)	Photo-cross-linker		Deiters / Chin	2011	Chem. Sci.	10.1039/c0sc00373e
MbPylRS / MbtRNAPyl	Wild type	N^{ε}-(*tert*-Butoxycarbonyl)-N^{ε}-methyl-*L*-lysine	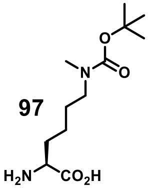 97	*E. coli*	Precursor to *N*-methyllysine		Chin	2009	J. Am. Chem. Soc.	10.1021/ja906603s
MbPylRS / MbtRNAPyl	L274A C313S Y349F	(3-(3-Methyl-*3H*-diazirine-3-yl)-propaminocarbonyl-N^{ε}-*L*-lysine (DiZPK)	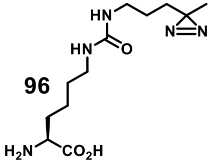 96	*E. coli*, Mammalian cells (CHO)	Photo-cross-linker	Much more efficient than pBpa.	Chang / Chen	2011	Nat. Chem. Biol	10.1038/nchembio.644
PylRS / tRNAPyl	Wild type	(3-(3-Methyl-*3H*-diazirine-3-yl)-propaminocarbonyl-N^{ε}-*L*-lysine (DiZPK)	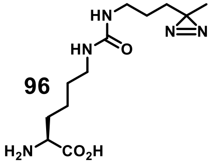 96	Enteropathogenic *E. coli* (EPEC), Shigella, Salmonella	Photo-cross-linker	Unconventional promoter needed in Salmonella	Chen	2011	J. Am. Chem. Soc	10.1021/ja209008w
MmPylRS / MmtRNAPyl	Y306M L309A C348T T364K		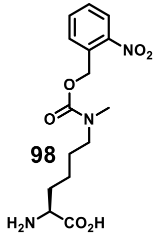 98	*E. coli*	Photocaged methyllysine		Liu	2010	Mol. Biosyst.	10.1039/c002155e

MbPylRS / MbtRNAPyl	Y271M L274G C313A	BCN (exo isomer)	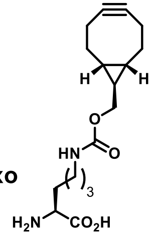 99exo	*E. coli*, mammalian cells (HEK)	Reactive handle for SPAAC		Chin	2012	J. Am. Chem. Soc.	10.1021/ja302832g
MmPylRS / MmtRNAPyl	Y306A Y384F	BCN (endo isomer)	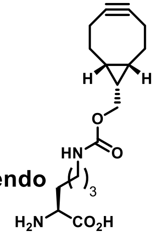 99endo	*E. coli,* mammalian cells (HeLa)	Reactive handle for SPAAC		Delft / Lemke	2012	ChemBioChem	10.1002/cbic.201200407
MmPylRS / MmtRNAPyl	Y306A Y384F	TCO	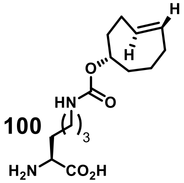 100	*E. coli,* mammalian cells (HeLa)	Reactive handle for IEDDA.		C. Schultz / Lemke	2012	Angew. Chem. Int. Edit.	10.1002/anie.201108231
MbPylRS / MbtRNAPyl	Y271A L274M C313A	TCO	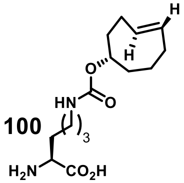 100	*E. coli*, mammalian cells (HEK)	Reactive handle for IEDDA		Chin	2012	J. Am. Chem. Soc.	10.1021/ja302832g
MmPylRS / MmtRNAPyl	Y306A Y384F		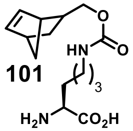 101	*E. coli,* mammalian cells (HeLa)	Reactive handle for IEDDA.		C. Schultz / Lemke	2012	Angew. Chem. Int. Edit.	10.1002/anie.201108231
MmPylRS / MmtRNAPyl	Y306A Y384F		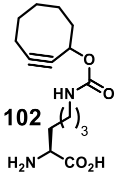 102	*E. coli,* mammalian cells (HeLa)	Reactive handle for SPAAC		C. Schultz / Lemke	2012	Angew. Chem. Int. Edit.	10.1002/anie.201108231

MbPylRS / MbtRNAPyl	L266M L270I Y271L L274A C313I	N^{ε}-(1-Methylcycloprop-2-enecarboxamido)lysine (CpK)	**103** 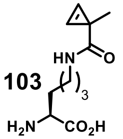	*E. coli*, mammalian cells (HEK)	Reactive handle for 'photo-click'	Stable to glutathione	Wang / Lin	2012	Angew. Chem. Int. Edit.	10.1002/anie.201205352
MbPylRS / MbtRNAPyl	A267S C313V M315F D344G	N^{ε}-*L*-Thiapropyl-*L*-lysine	**104** 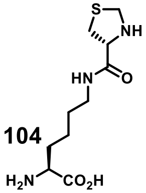	*E. coli*	Precursor to NCL reactive handle		Chin	2011	J. Am. Chem. Soc	10.1021/ja203111c
MmPylRS / MmtRNAPyl	Wild Type		**105** 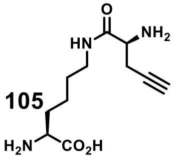	*E. coli*	Reactive handle for azide-alkyne cycloaddition		Chan	2010	Chem. Asian J.	10.1002/asia.201000205
MmPylRS / MmtRNAPyl	Wild Type		**106** 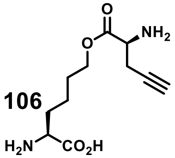	*E. coli*	Reactive handle for azide-alkyne cycloaddition that can subsequently be hydrolysed		Chan	2013	Chembiochem	10.1002/cbic.201300124
MmPylRS / MmtRNAPyl	Y384F	N^{ε} -Allyloxycarbonyl-l-lysine (AlocLys)	**107** 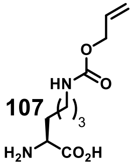	*E. coli*			Yokoyama	2008	Chem. Bio.	10.1016/j.chembiol.2008.10.004
MbPylRS / MbtRNAPyl	L274A C313V Y349F	N^{ε} -Allyloxycarbonyl-l-lysine (AlocLys)	**107** 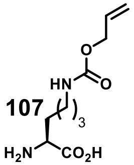	*E. coli*			Liu / Chen	2012	Chem.Sci.	10.1039/c2sc20433a

MbPylRS / MbtRNAPyl	L274A C313V Y349F	BlocLys	108 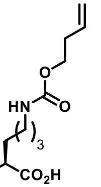	*E. coli*	Reactive handle for thiol-ene reaction	Used for double incorporation/ dual labelling	Liu / Chen	2012	Chem.Sci.	10.1039/c2sc20433a
MbPylRS / MbtRNAPyl	L274A C313V Y349F	PlocLys	109 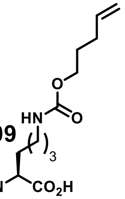	*E. coli*			Liu / Chen	2012	Chem.Sci.	10.1039/c2sc20433a
MmPylRS / MmtRNAPyl	Wild type	Boc-LysOH	110 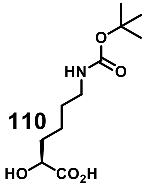	*E. coli*			Sakamoto / Yokoyama	2009	J. Mol. Biol.	10.1016/j.jmb.2008.11.059
MbPylRS / MbtRNAPyl	L274A C313V Y349F	Boc-LysOH	110 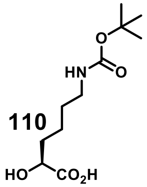		Hydroxy acid to introduce ester linkages into protein backbone		Liu / Chen	2012	ACS Chem. Biol.	10.1021/cb300020s
MmPylRS / MmtRNAPyl	L301M Y306L L309A C348F Y384W	N^{ε}-Acryloyl-*L*-lysine	111 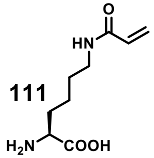	*E. coli*	Reactive handle for 1,4-cycloadditions, radical copolymerisation and 1,3-dipolar cycloaddition	Synthetase also incorporates propyl butyl and crotyl analogues	Liu	2013	ACS Chem. Biol.	10.1021/cb400267m
MbPylRS / MbtRNAPyl	D76G L266M L270I Y271F L274A C313F	N^{ε}-Acryloyl-*L*-lysine	111 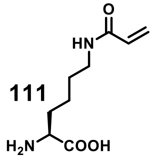	*E. coli*, Mammalian CHO-cells, plant (A. thaliana)	Reactive handle for 'photo-click'	First UAA incorporation in a plant	Wang	2013	Angew. Chem. Int. Edit.	10.1002/anie.201303477

MbPylRS / MbtRNAPyl	L274A C313V Y349F		112 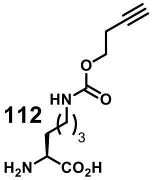	*E. coli*			Guo	2013	Org. Biomol. Chem.	10.1039/c3ob27116a
MbPylRS / MbtRNAPyl	L274A C313V Y349F		113 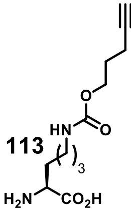	*E. coli*	Reactive handle for thiol-yne reaction	Most efficiently incorporated of the 3 alkyne analogues described	Guo	2013	Org. Biomol. Chem.	10.1039/c3ob27116a
MbPylRS / MbtRNAPyl	L274A C313S Y349F		113 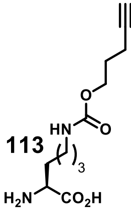	Enteropathogenic *E. coli* (EPEC), Shigella, Salmonella	Reactive handle for *in vitro* and *in vivo* Sonogashira coupling	Not incorporated by wtPylRS	Chen	2013	J. Am. Chem. Soc.	10.1021/ja402424j
MbPylRS / MbtRNAPyl	L274A C313S Y349F		114 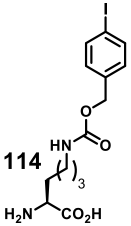	Enteropathogenic *E. coli* (EPEC), Shigella, Salmonella	Reactive handle for *in vitro* and *in vivo* Sonogashira coupling	Not incorporated by wtPylRS	Chen	2013	J. Am. Chem. Soc.	10.1021/ja402424j
MmPylRS / MmtRNAPyl	Wild type	N^{ε}-(Spiro[2.3]hex-1-ene-5-methoxycarbonyl)-*L*-lysine	115 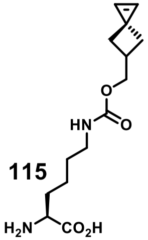	*E. coli*	Reactive handle for photo-click chemistry		Lin	2014	J. Am. Chem. Soc.	10.1021/ja5012542

MmPylRS / MmtRNAPyl	Y306G, Y384F	pNO_2ZLys	116 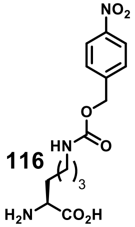	*E. coli*, mammalian HEK and CHO-cells	Photo-cross-linker		Sakamoto / Yokoyama	2012	Mol. Biosyst.	10.1039/c2mb05321g
MmPylRS / MmtRNAPyl	Y306G, Y384F	TmdZLys	117 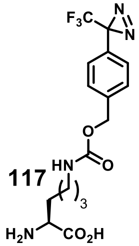	*E. coli*, mammalian-cells (HEK, CHO)	Photo-cross-linker		Sakamoto / Yokoyama	2012	Mol. Biosyst.	10.1039/c2mb05321g
MbPylRS / MbtRNAPyl	D76G L266V L270I Y271F L274A C313F	2-Amino-8-oxononanoic acid (KetoK)	118 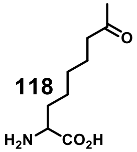	*E. coli*			Liu	2010	Bioorg. Med. Chem. Lett.	10.1016/j.bmcl.2009.12.077
MmPylRS / MmtRNAPyl	Wild Type	N^{ε}-Propionyl-*L*-lysine (Kpr)	119 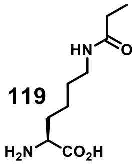	*E. coli*	Natural PTM	Nicotinamide necessary to inhibit enzyme responsible of decrotylation	Carell	2013	Chem. Commun.	10.1039/C2CC37836A
MmPylRS / MmtRNAPyl	Wild Type	N^{ε}-Butyryl-*L*-lysine (Kbu)	120 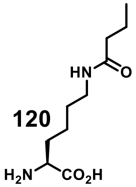	*E. coli*	Natural PTM	Nicotinamide necessary to inhibit enzyme responsible of decrotylation	Carell	2013	Chem. Commun.	10.1039/C2CC37836A

MmPylRS / MmtRNAPyl	L274A<br>C313F<br>Y349F	N^{ε}-Crotonyl-*L*-lysine (Kcr)	121 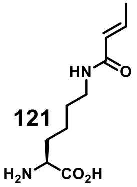	*E. coli*, mammalian cells (HEK293T)	Natural PTM	Nicotinamide necessary to inhibit enzyme responsible of decrotylation	Schultz	2012	Angew. Chem. Int. Edit.	10.1002/anie.201203349
MmPylRS / MmtRNAPyl	Wild Type	N^{ε}-Crotonyl-*L*-lysine (Kcr)	121 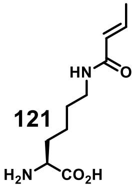	*E. coli*	Natural PTM	Nicotinamide necessary to inhibit enzyme responsible of decrotylation	Carell	2013	Chem. Commun.	10.1039/C2CC37836A
MmPylRS / MmtRNAPyl	Y306A<br>Y384F		122 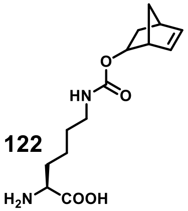	*E. coli*, mammalian cells (HeLa)	Reactive handle for IEDDA.		C. Schultz / Lemke	2012	Angew. Chem. Int. Edit.	10.1002/anie.201108231
MbPylRS / MbtRNAPyl	Wild type	N^{ε}-5-Norbornene-2-yloxycarbonyl-*L*-lysine	122 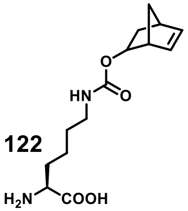	*E. coli*	Reactive handle for IEDDA		Chin	2012	Nat. Chem.	10.1038/nchem.1250
MmPylRS / MmtRNAPyl	A302T<br>N346G<br>C348T<br>V401I<br>W417Y	*L*-3-(2-Naphtyl)alanine (Nap)	128 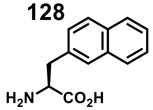	*E. coli*		Improved evolution strategy (small-intelligent mutagenesis)	Wang	2013	ChemBioChem	10.1002/cbic.201300400
MbPylRS / MbtRNAPyl (*E. coli*)<br>Or MmPylRS / MmtRNAPyl (mammalian)	L270I<br>Y271F<br>L274G<br>C313F<br>Y349F		129 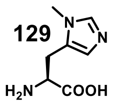	*E. coli*, mammalian cells (HEK)			Schultz	2014	ACS Chem. Biol.	10.1021/cb500032c

MbPylRS / MbtRNAPyl (*E. coli*) Or MmPylRS / MmtRNAPyl (mammalian)	L270I Y271F L274G C313F Y349F		130 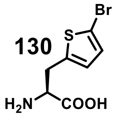	*E. coli*, mammalian cells (HEK)			Schultz	2014	ACS Chem. Biol.	10.1021/cb500032c
MbPylRS / MbtRNAPyl (*E. coli*) Or MmPylRS / MmtRNAPyl (mammalian)	L270I Y271F L274G C313F Y349F		131 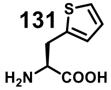	*E. coli*, mammalian cells (HEK)			Schultz	2014	ACS Chem. Biol.	10.1021/cb500032c
MbPylRS / MbtRNAPyl (*E. coli*) Or MmPylRS / MmtRNAPyl (mammalian)	L270I Y271F L274G C313F Y349F		132 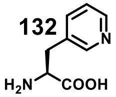	*E. coli*, mammalian cells (HEK)			Schultz	2014	ACS Chem. Biol.	10.1021/cb500032c
MbPylRS / MbtRNAPyl (*E. coli*) Or MmPylRS / MmtRNAPyl (mammalian)	L270I Y271F L274G C313F Y349F		133 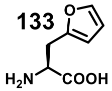	*E. coli*, mammalian cells (HEK)			Schultz	2014	ACS Chem. Biol.	10.1021/cb500032c
MmPylRS / MmtRNAPyl	N346A C348A		135 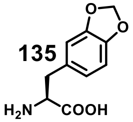	*E. coli*		Over 40 substrates for single synthetase mutant	Liu	2014	Chem. Commun.	10.1039/c3cc49068h
MmPylRS / MmtRNAPyl	N346A C348A		136 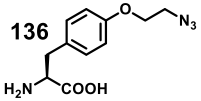	*E. coli*		Over 40 substrates for single synthetase mutant	Liu	2014	Chem. Commun.	10.1039/c3cc49068h
MmPylRS / MmtRNAPyl	N346A C348A		137 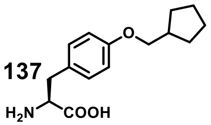	*E. coli*		Over 40 substrates for single synthetase mutant	Liu	2014	Chem. Commun.	10.1039/c3cc49068h
MmPylRS / MmtRNAPyl	N346A C348A		138 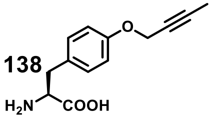	*E. coli*		Over 40 substrates for single synthetase mutant	Liu	2014	Chem. Commun.	10.1039/c3cc49068h

MmPylRS / MmtRNAPyl	Y306A L309M Y384F		139 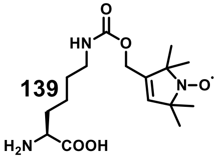	*E. coli*	Spin label		Drescher / Summerer	2014	J. Am. Chem. Soc.	10.1021/ja4115 35q
MmPylRS / MmtRNAPyl	Y306A Y384F	N^{ε}-[2-(furan-2-yl)ethoxy]carbonyl lysine	140 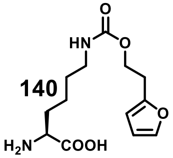	*E. coli*	Reactive handle for cross-linking DNA with red light		Summerer	2013	Angew. Chem. Int. Edit.	10.1002/anie.2 01300754
MbPylRS / MbtRNAPyl (*E. coli*) Or MmPylRS / MmtRNAPyl (mammalian)	N311Q C313A V366M		141 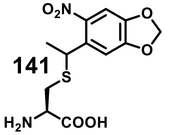	*E. coli*, mammalian cells (HEK)	Photo-caged cysteine		Chin	2014	J. Am. Chem. Soc.	10.1021/ja4121 91m
MmPylRS / MmtRNAPyl	N346A C348A		142 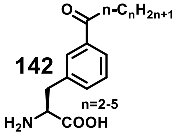	*E. coli*		Over 40 substrates for single synthetase mutant	Liu	2014	Chem. Commun.	10.1039/c3cc49 068h
MmPylRS / MmtRNAPyl	A302T N346A C348A Y384F W417T		143 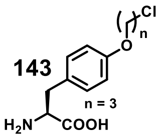	*E. coli*			Wang	2014	Angew. Chem. Int. Edit.	10.1002/anie.2 01308794
MmPylRS / MmtRNAPyl	A302T N346A C348A Y384F W417T		144 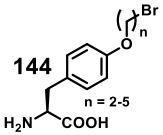	*E. coli*	Reactive handle for cross linking proteins at cysteine		Wang	2014	Angew. Chem. Int. Edit.	10.1002/anie.2 01308794
MmPylRS / MmtRNAPyl	A302T N346A C348A Y384F W417T		145 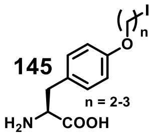	*E. coli*			Wang	2014	Angew. Chem. Int. Edit.	10.1002/anie.2 01308794
MmPylRS / MmtRNAPyl	N346A C348A	3-Formyl-*L*-phenylalanine	146 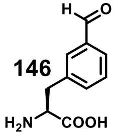	*E. coli*	Reactive handle for reaction with hydroxylamine dyes		Liu	2014	Chem. Commun.	10.10.1039/c4c c02000f

MmPylRS / MmtRNAPyl	N346A C348A	3-Ethynyl-*L*-phenylalanine	147 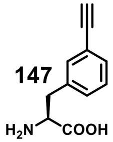	*E. coli*	Reactive handle for azide-alkyne cycloaddition or Sonogashira coupling		Liu	2013	ACS Chem. Biol.	10.1021/cb300512r
MmPylRS / MmtRNAPyl	N346A C348A	2-Chloro-*L*-phenylalanine	149 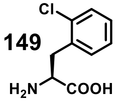	*E. coli*, mammalian cells		Over 40 substrates for single synthetase mutant	Liu	2014	ACS Chem. Biol.	10.1021/cb400917a
MmPylRS / MmtRNAPyl	N346A C348A	2-Bromo-*L*-phenylalanine	150 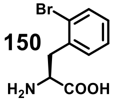	*E. coli*, mammalian cells		Over 40 substrates for single synthetase mutant	Liu	2014	ACS Chem. Biol.	10.1021/cb400917a
MmPylRS / MmtRNAPyl	N346A C348A	2-Iodo-*L*-phenylalanine	151 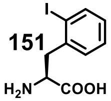	*E. coli*, mammalian cells		Over 40 substrates for single synthetase mutant	Liu	2014	ACS Chem. Biol.	10.1021/cb400917a
MmPylRS / MmtRNAPyl	N346A C348A	2-Methyl-*L*-phenylalanine	152 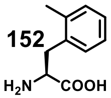	*E. coli*, mammalian cells		Over 40 substrates for single synthetase mutant	Liu	2014	ACS Chem. Biol.	10.1021/cb400917a
MmPylRS / MmtRNAPyl	N346A C348A	2-Methoxy-*L*-phenylalanine	153 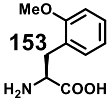	*E. coli*, mammalian cells		Over 40 substrates for single synthetase mutant	Liu	2014	ACS Chem. Biol.	10.1021/cb400917a
MmPylRS / MmtRNAPyl	N346A C348A	2-Nitro-*L*-phenylalanine	154 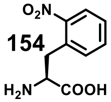	*E. coli*, mammalian cells		Over 40 substrates for single synthetase mutant	Liu	2014	ACS Chem. Biol.	10.1021/cb400917a
MmPylRS / MmtRNAPyl	N346A C348A	2-Cyano-*L*-phenylalanine	155 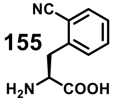	*E. coli*, mammalian cells		Over 40 substrates for single synthetase mutant	Liu	2014	ACS Chem. Biol.	10.1021/cb400917a
MmPylRS / MmtRNAPyl	N346A C348A		156 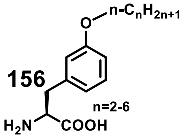	*E. coli*		Over 40 substrates for single synthetase mutant	Liu	2014	Chem. Commun.	10.1039/c3cc49068h

MmPylRS / MmtRNAPyl	N346A C348A	3-Nitro-*L*-phenylalanine	157 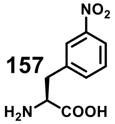	*E. coli*			Liu	2013	ACS Chem. Biol.	10.1021/cb300512r
MmPylRS / MmtRNAPyl	N346A C348A	3-Cyano-*L*-phenylalanine	158 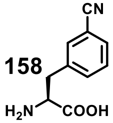	*E. coli*			Liu	2013	ACS Chem. Biol.	10.1021/cb300512r
MmPylRS / MmtRNAPyl	N346A C348A	3-Azido-*L*-phenylalanine	159 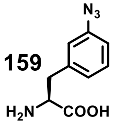	*E. coli*	Reactive handle for azide-alkyne cycloaddition		Liu	2013	ACS Chem. Biol.	10.1021/cb300512r
MmPylRS / MmtRNAPyl	N346A C348A	3-Fluoro-*L*-phenylalanine	160 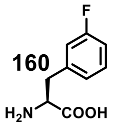	*E. coli*			Liu	2013	ACS Chem. Biol.	10.1021/cb300512r
MmPylRS / MmtRNAPyl	N346A C348A	3-Chloro-*L*-phenylalanine	161 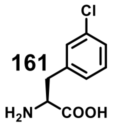	*E. coli*			Liu	2013	ACS Chem. Biol.	10.1021/cb300512r
MmPylRS / MmtRNAPyl	N346A C348A	3-Bromo-*L*-phenylalanine	162 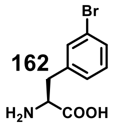	*E. coli*			Liu	2013	ACS Chem. Biol.	10.1021/cb300512r
MmPylRS / MmtRNAPyl	N346A C348A	3-Iodo-*L*-phenylalanine	163 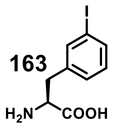	*E. coli*			Liu	2013	ACS Chem. Biol.	10.1021/cb300512r
MmPylRS / MmtRNAPyl	N346A C348A	3-Methyl-*L*-phenylalanine	164 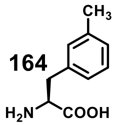	*E. coli*			Liu	2013	ACS Chem. Biol.	10.1021/cb300512r
MmPylRS / MmtRNAPyl	N346A C348A	3-Trifluoromethyl-*L*-phenylalanine	165 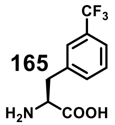	*E. coli*	^{19}F Probe for protein NMR		Liu	2013	ACS Chem. Biol.	10.1021/cb300512r

MmPylRS / MmtRNAPyl	N346A C348A	3-Methoxy-*L*-phenylalanine	166 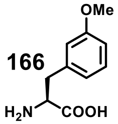	*E. coli*			Liu	2013	ACS Chem. Biol.	10.1021/cb300512r
MmPylRS / MmtRNAPyl	N346A C348A		167 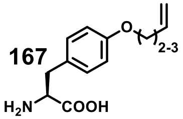	*E. coli*	Speculated bioorthogonal reactive handle (CuAAC)		Liu	2012	J. Am. Chem. Soc.	10.1021/ja211972x
**Misc. RS**										
M. acetivorans TyrRS/tRNA	Y33A H71A Q113I D162E I163L	3-Iodo-*L*-tyrosine	4 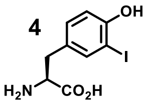	*E. coli*			Nishikawa	2013	J. Biochem.	10.1093/jb/mvs153
M. acetivorans TyrRS/tRNA	Y33A H71A Q113I D162E I163L	3-Azido-*L*-tyrosine	75 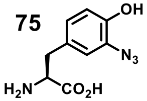	*E. coli*	Photo-cross-linker		Nishikawa	2013	J. Biochem.	10.1093/jb/mvs153
P. horikoshii Ph$tRNA_{UCCU}$/Lys-RS	E41I Y268S	Homoglutamine	123 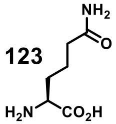	*E. coli*		Can be incorporated via quadruplet suppression and used in conjunction with amber suppression	Schultz	2004	Proc. Natl. Acad. Sci.	10.1073/pnas.0401517101
M. maripaludis SepRS / MjtRNACys	tRNA: C20U G34C C35U	Phosphoserine	168 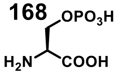	*E. coli*	Natural post-translational modification		Söll	2011	Science	10.1126/science.1207203
**QUADRUPLET**										
MjTyrRS / $tRNATyr_{UCCU}$	Ribo-Q1: A1196G A1197G; RS: Y230 C231K P232K H283Q D286S	*p*-Azido-*L*-phenylalanine (AzPhe)	6 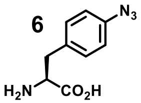	*E. coli*	Double substitution with N6-[(2-propynyloxy)carbonyl]-*L*-lysine for protein cyclisation	Orthogonal to MbPylRS / MbtRNAPyl Use of orthogonal ribosome ribo-Q1	Chin	2010	Nature	10.1038/nature08817

MmPylRS / $tRNAPyl_{UCCU}$	tRNA: A28G C29A A35C U36C C38U G39A **OR** U25G A28G A35U U36C C38G G39C. PylRS: Y384F	N^{ε}-(*tert*-Butoxycarbonyl)-*L*-lysine (BocLys)	77 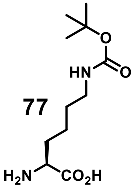	*E. coli*			Guo	2013	ACS Chem. Biol.	10.1021/cb400 1662
MmPylRS / $tRNAPyl_{UCCU}$	tRNA: U25G A28G A35U U36C C38G G39C PylRS: Y384F	N^{ε}-(*tert*-Butoxycarbonyl)-*L*-lysine (BocLys)	77 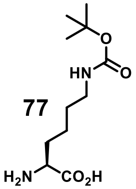	Mammalian cells (HEK 293T)			Guo	2013	ACS Chem. Biol.	10.1021/cb400 1662
MmPylRS / $tRNAPyl_{UCUA}$	tRNA: A31G A38U U39C C40U	N^{ε}-(*tert*-Butoxycarbonyl)-*L*-lysine (BocLys)	77 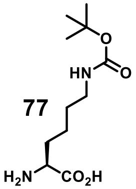	*E. coli*			Chin	2014	Nat. Chem.	10.1038/nchem .1919
MmPylRS / $tRNAPyl_{UACU}$	tRNA: G29U A31G A38U U39C C41U	N^{ε}-[(2-Propynyloxy)carbonyl] -*L*-lysine	86 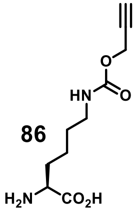	*E. coli*			Chin	2014	Nat. Chem.	10.1038/nchem .1919
MmPylRS / $tRNAPyl_{UCCU}$	tRNA: G29C G30C A31U C40A C41G		107 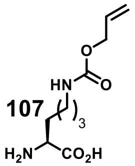	*E. coli*			Chin	2014	Nat. Chem.	10.1038/nchem .1919

MmPylRS / tRNAPyl$_{UACU}$	tRNA: G29U<br>A31G<br>A38U<br>U39C<br>C41U<br>PylRS: Y384F<br>Y306G<br>I405R		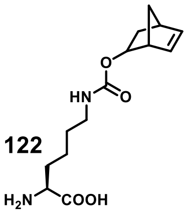 122	*E. coli*			Chin	2014	Nat. Chem.	10.1038/nchem.1919
Pyrococcus horikoshii LysRS / tRNALys$_{UCCU}$	tRNA: G1U<br>C4U<br>A37C<br>G69A<br>C71U<br>C72A.<br>RS: E41I<br>Y286S<br>truncation after S357 for decreased toxicity	Homoglutamine	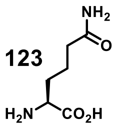 123	*E. coli*		Orthogonal to MjTyrRS / MjtRNATyr mutant for OMe-Tyr. Simultaneous incorporation *via* amber and quadruplet suppression. Natural ribosome used.	Schultz	2004	Proc. Natl. Acad. Sci.	10.1073/pnas.0401517101
**ANIMALS**										
E. coli TyrRS / tRNATyr	Y37T<br>D182T<br>L183M<br>D265R	*O*-Methyl-*L*-tyrosine	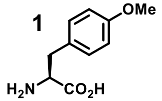 1	*C.elegans*	Labelling of muscles (global)		Wang	2012	ACS Chem. Biol.	10.1021/cb200542j
E. coli TyrRS / Yam (suppressor tRNA derived Bacillus stearothermophilus)	Y37G<br>D182G<br>L186A	*p*-Benzoyl-*L*-phenylalanine (*p*Bpa)	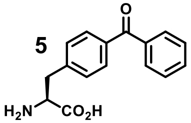 5	*Xenopus laevis* oocytes			Paoletti	2013	ChemBioChem	10.1002/cbic.201200515
E. coli TyrRS / Yam (suppressor tRNA derived Bacillus stearothermophilus)	Y37L<br>D182S<br>F183M<br>L186A	*p*-Azido-*L*-phenylalanine (AzPhe)	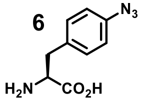 6	*Xenopus laevis* oocytes	Photo-cross-linker	Leaky suppression	Paoletti	2013	ChemBioChem	10.1002/cbic.201200515
E. coli LeuRS / tRNALeu	M40A<br>L41N<br>T252A<br>Y499I<br>Y527G<br>H537T	2-Amino-3-(5-(dimethylamino)naphthalene-1-sulfonamido)propanoic acid (DanAla)	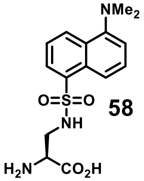 58	*C.elegans*	Labelling of muscles (global)	Dipeptide (DanAla-Ala) required for uptake and bioavailability	Wang	2012	ACS Chem. Biol.	10.1021/cb200542j

MmPylRS / MmtRNAPyl	Wild type	N^{ε}-(*tert*-Butoxycarbonyl)-*L*-lysine (BocLys)	77 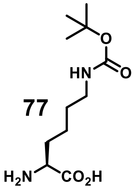	*C.elegans*		Extra-chromosomal array (low stability), biolistic bombardment. Scattered in different places in animal.	Chin	2011	J. Am. Chem. Soc.	10.1021/ja2054034
MmPylRS / MmtRNAPyl	Wild type	N^{ε}-(*tert*-Butoxycarbonyl)-*L*-lysine (BocLys)	77 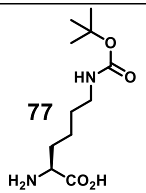	*D. melanogaster* (cells, embryos and adult flies)		Tissue specific	Chin	2012	Nat. Chem. Biol.	10.1038/nchembio.1043
MmPylRS / MmtRNAPyl	Wild type	N^{ε}-[(2-Propynyloxy)carbonyl]-*L*-lysine	86 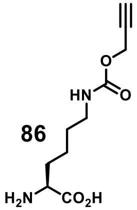	*C.elegans*	Reactive handle for azide-alkyne cycloaddition	Extra-chromosomal array (low stability), biolistic bombardment. Scattered in different places in animal.	Chin	2011	J. Am. Chem. Soc.	10.1021/ja2054034
MmPylRS / MmtRNAPyl	Wild type	N^{ε}-[(2-Propynyloxy)carbonyl]-*L*-lysine	86 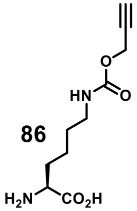	*D. melanogaster* (cells, embryos)			Chin	2012	Nat. Chem. Biol.	10.1038/nchembio.1043
MmPylRS / MmtRNAPyl	Wild type		122 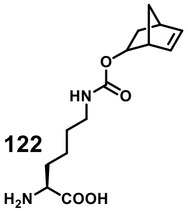	*D. melanogaster* cells	Reactive handle for IEDDA		Chin	2012	Nat. Chem. Biol.	10.1038/nchembio.1043
